# Pi USB Cam: A Simple and Affordable DIY Solution That Enables High-Quality, High-Throughput Video Capture for Behavioral Neuroscience Research

**DOI:** 10.1523/ENEURO.0224-22.2022

**Published:** 2022-09-23

**Authors:** Shikun Hou, Elizabeth J. Glover

**Affiliations:** Center for Alcohol Research in Epigenetics, Department of Psychiatry, University of Illinois at Chicago, Chicago, IL 60612

**Keywords:** behavior, plug-and-play, recording, scalable

## Abstract

Video recording is essential for behavioral neuroscience research, but the majority of available systems suffer from poor cost-to-functionality ratio. Commercial options frequently come at high financial cost that prohibits scalability and throughput, whereas DIY solutions often require significant expertise and time investment unaffordable to many researchers. To address this, we combined a low-cost Raspberry Pi microcomputer, DIY electronics peripherals, freely available open-source firmware, and custom 3D-printed casings to create Pi USB Cam, a simple yet powerful and highly versatile video recording solution. Pi USB Cam is constructed using affordable and widely available components and requires no expertise to build and implement. The result is a system that functions as a plug-and-play USB camera that can be easily installed in various animal testing and housing sites and is readily compatible with popular behavioral and neural recording software. Here, we provide a comprehensive parts list and step-by-step instructions for users to build and implement their own Pi USB Cam system. In a series of benchmark comparisons, Pi USB Cam was able to capture ultra-wide fields of view of behaving rats given limited object distance and produced high image quality while maintaining consistent frame rates even under low-light and no-light conditions relative to a standard, commercially available USB camera. Video recordings were easily scaled using free, open-source software. Altogether, Pi USB Cam presents an elegant yet simple solution for behavioral neuroscientists seeking an affordable and highly flexible system to enable quality video recordings.

## Significance Statement

Video capture is increasingly necessary for neuroscience research where neural and behavioral data are synchronized to reveal correlative and causal relationships. This relies on a recording system that can capture quality videos without significant alterations to preexisting experimental conditions (e.g., lighting, space, etc.), enables easy online and offline analysis by commonly used software, and offers high scalability to increase throughput. However, the high cost and poor flexibility of commercially available options leave the role of an ideal video recording system unfulfilled. Here, we present a DIY video recording solution that combines affordable electronics hardware and custom 3D-printed components with sophisticated open-source software to make a simple, yet powerful USB camera that satisfies almost any recording need.

## Introduction

Video recorded data are a crucial component of behavioral neuroscience research. While a number of commercially available recording solutions exist, few, if any, satisfy all or most of the needs of a typical laboratory. Many behavioral assays, particularly those intended for use with nocturnal species, are conducted under low-light or no-light conditions to mimic the portion of the circadian cycle during which animals are the most active. Therefore, an ideal video recording system enables video acquisition under both ambient and infrared (IR) illumination. Moreover, spatial limitations imposed by testing apparatuses frequently require capture of a large field of view from a relatively short object distance. Although some commercially available options designed specifically for behavioral neuroscience research meet these criteria, they are often expensive and unique to a specific testing apparatus limiting scalability and flexibility of use. Given these limitations, researchers frequently repurpose commercial USB webcams for video acquisition because of their affordability and accessibility. However, webcams are rarely intended for recording under low-light to no-light conditions, nor for capturing a large field of view under circumstances when subjects are at a relatively short distance from the camera.

DIY solutions that make use of low-cost, single board microcomputers such as Raspberry Pi have gained the attention of behavioral neuroscientists as laboratories attempt to address the shortcomings of existing commercially available solutions. For example, Singh et al. (2019) developed a powerful web-based interface for long duration, remote video recording and streaming. However, lack of active maintenance and development quickly rendered it obsolete as the current generation of Raspberry Pi board (4B) no longer supports the legacy OS (Raspbian Stretch) for which it was developed. Recently developed solutions that use the Raspberry Pi Camera in its most basic form to acquire video are less demanding in terms of debugging and updating (Saxena et al., 2018; Weber and Fisher, 2019; Clemensson et al., 2020; Centanni and Smith, 2021). However, these out-of-the-box options suffer from limited functionality, often lacking the capacity to live preview and record at the same time, or interface with closed-loop behavioral control systems. As a result, DIY solutions frequently require a degree of programming knowledge to properly configure and adapt the system to specific research needs thereby limiting broad application of such systems by novice users. Thus, drawbacks associated with both commercially available and DIY video capture solutions pose significant limitations to easy acquisition of video recorded behavioral data.

To address this gap, we designed a versatile, low-cost, DIY video recording solution that requires no specialized expertise or equipment. Pi USB Cam is low-/no-light compatible and accepts a variety of wide-angle lenses. The system utilizes open-source, actively maintained software that enables true plug-and-play capability. Combined with custom 3D-printed components and freely available open-source video acquisition software, this system offers highly versatile implementation and scalability across numerous behavioral testing conditions. Here, we provide detailed step-by-step instructions for hardware and software setup and demonstrate its ease of use and superiority in terms of field of view and low-light recording over commercial counterparts.

## Materials and Methods

### Build the camera

Pi USB Cam is comprised of a Raspberry Pi board (Raspberry Pi, Cambridge) and a wide-angle day-night vision camera (Arducam Technology Co, Limited). This camera comes equipped with a motorized IR-CUT filter that is automatically triggered based on ambient light intensity to accommodate for both bright-light and low-/no-light recording, as well as an out-of-the-box 170° [diagonal field of view (DFOV)] × 140° [horizontal FOV (HFOV)] fisheye lens. The camera uses a 5MP OV5647 sensor, which can support up to 30 frames per second (FPS) at 1080p when used with the suggested firmware. Additionally, the camera comes attached with two 850-nm IR LEDs to facilitate image acquisition in low-/no-light conditions.

We selected the Raspberry Pi four Model B to run the camera, which was the latest release of the main product line at the time of publication. This model and the more affordable and compact Raspberry Pi Zero are readily supported by *Show-me webcam*, an open-source firmware that enables a Raspberry Pi connected camera to be booted as a simple USB camera. While both models are compatible as the base hardware for building a Pi USB Cam, we chose Pi 4B over Zero because of its added processing power, which is significantly more advantageous if the Pi board were to be repurposed for GUI applications. Of note, while the Raspberry Pi Zero 2 was recently released, it is not yet supported by *Show-me webcam*, although this is likely to change in near future. Legacy boards like Pi 3+ and earlier releases that are no longer widely accessible for purchase are not supported by *Show-me webcam*.

A complete list of store-bought components is provided in Table 1 with a more comprehensive list including alternative options provided in Extended Data Table 1-1.

**Table 1:** Store-bought components list

Product	Vendor	Catalog number	Price	Comment
RASPBERRY PI BOARD:				
Raspberry Pi 4 Model B (1/2/4/8GB)	Anywhere	n/a	$35–75	Can be purchased as a kit with accessories at a higher cost
CAMERA MODULE:				
Arducam Wide Angle Day-Night Vision for Raspberry Pi Camera [FOV: 170° (D) × 140° (H)]	UCTRONICS https://bit.ly/39PKPH8	B003507	$32.99	
ACCESSORIES:				
MicroSD Card (16GB)	PiShop.us https://bit.ly/38Nc45c	936	$7.95	Any microSD card with a minimum storage space of 64MB would work
Raspberry Pi 4 Compatible Heavy-Duty Aluminum Alloy Case with Pre-installed Ready to Connect Fan	Vilros https://bit.ly/3GnbeIQ	VILP015	$14.99	Any case with camera ribbon cable and GPIO access would work; Metal cases with preinstalled cooling fan would be preferred to prevent overheating of the Raspberry Pi board
Standard USB to USB-C Cable (6 ft)	Vilros https://bit.ly/3alpw0C	VILP103	$4.99	Any USB-C type cable capable of data transferring
Jumper Cable Pin Header Connector Housing Assortment Kit	Amazon https://amzn.to/3z3Z0mu	B077X8XV2J	$12.98	For wiring the IR LED boards to use them independently of the camera board
Ring Terminal, Non Insulated, 22–16 Wire Size, #4 Stud Size	Amazon https://amzn.to/3LKqUH9	B005GDFMSG	$11.23	For wiring the IR LED boards to use them independently of the camera board
Red Black 2 Pin Wires 22 AWG (100 ft)	Amazon https://amzn.to/3wJhnvN	B0793N3WZZ	$18.99	For wiring the IR LED boards to use them independently of the camera board
Heat Shrink Tubing Kit	Jameco https://bit.ly/3NxH5bX	2095963	$17.95	For wiring the IR LED boards to use them independently of the camera board
Crimping Tool Set	Amazon https://amzn.to/38Fy1mR	B0045CUMLQ	$66.74	For wiring the IR LED boards to use them independently of the camera board
Wire Stripper	Grainger https://bit.ly/3MOaMps	1XFZ6	$34.25	For wiring the IR LED boards to use them independently of the camera board
4-Port USB PCIe Card	CDW https://bit.ly/3aoX6Tz	6409687	$114.99	For expanding the total USB bandwidth of the host computer during multicamera recording

Vendor, catalog number, and price for all store-bought components used in current build. See Extended Data Table 1-1 for a comprehensive list of parts plus alternative options and additional accessories. See Extended Data Table 1-2 for cost estimates for complete build. n/a: not applicable.

#### Step-by-step instructions

##### Software installation

**Figure 1.**
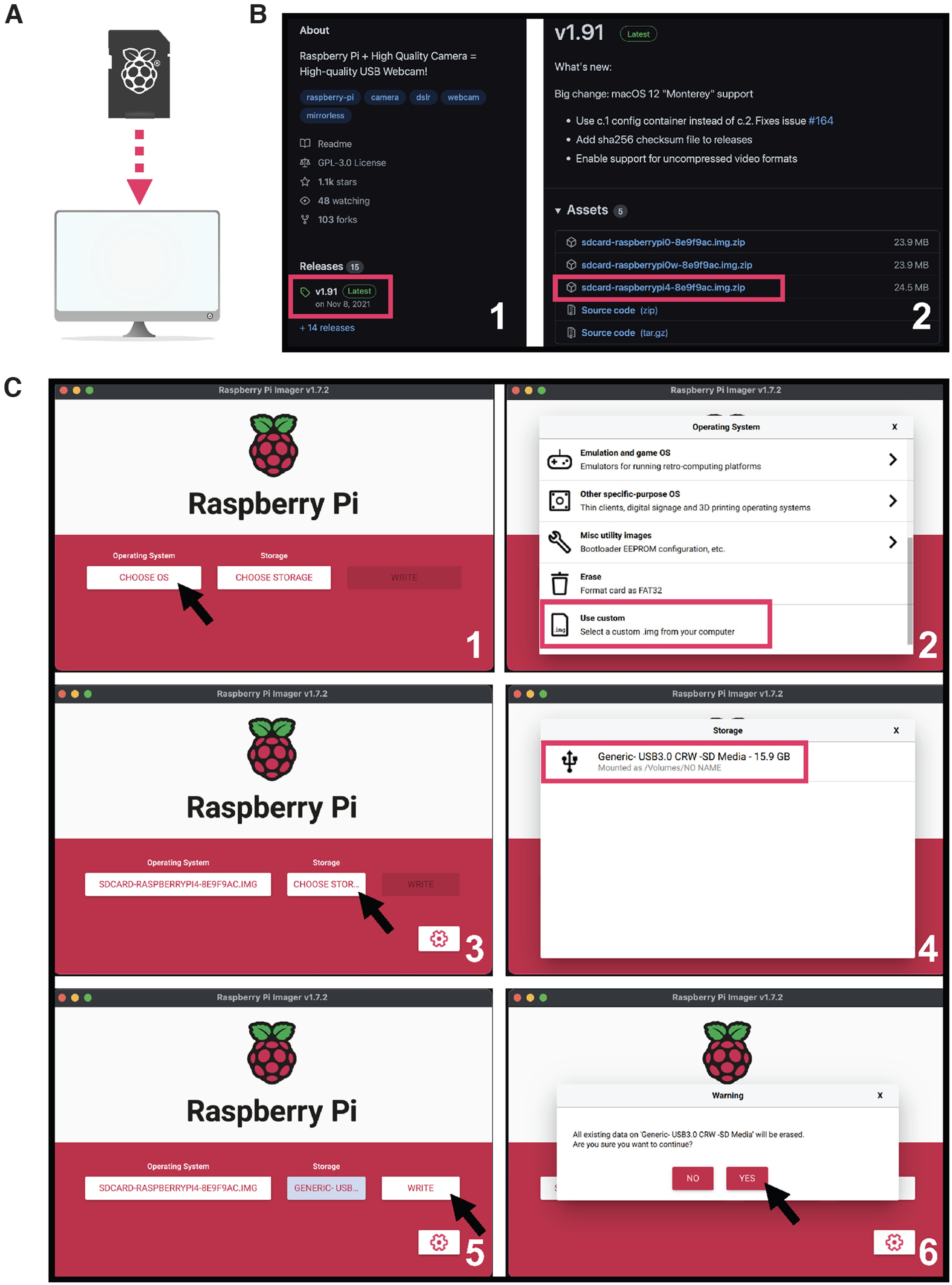
Camera software setup. ***A***, Insert a clean micro-SD card into a computer. ***B***, Download the latest release of the *Show-me webcam* image file from its official GitHub page. ***C***, Install the *Show-me webcam* image file on the micro-SD card using the official Raspberry Pi Imager. Image source: https://github.com/raspberrypilearning/components.

1. Insert a clean micro-SD card with a minimum storage of 64MB into your computer (Fig. 1*A*).
2. Download the latest release of the *Show-me webcam* image file to your computer from the developer’s GitHub page https://github.com/showmewebcam/showmewebcam (Fig. 1*B*). Make note to download the image file corresponding to the appropriate Raspberry Pi board model.
3. Download, install, and launch the official Raspberry Pi Imager from https://www.raspberrypi.com/software/ (Fig. 1*C*). Inside the imager’s main interface, click on **CHOOSE OS** followed by **Use custom** to locate the image file you just downloaded (Fig. 1*C1*,*C2*). Then, click on **CHOOSE STORAGE** and select the clean micro-SD card (Fig. 1*C3*,*C4*). Lastly, click on **WRITE** followed by **YES** on the pop-up warning to flash the *Show-me webcam* image file into the micro-SD card (Fig. 1*C5*,*C6*).

##### Hardware assembly

**Figure 2.**
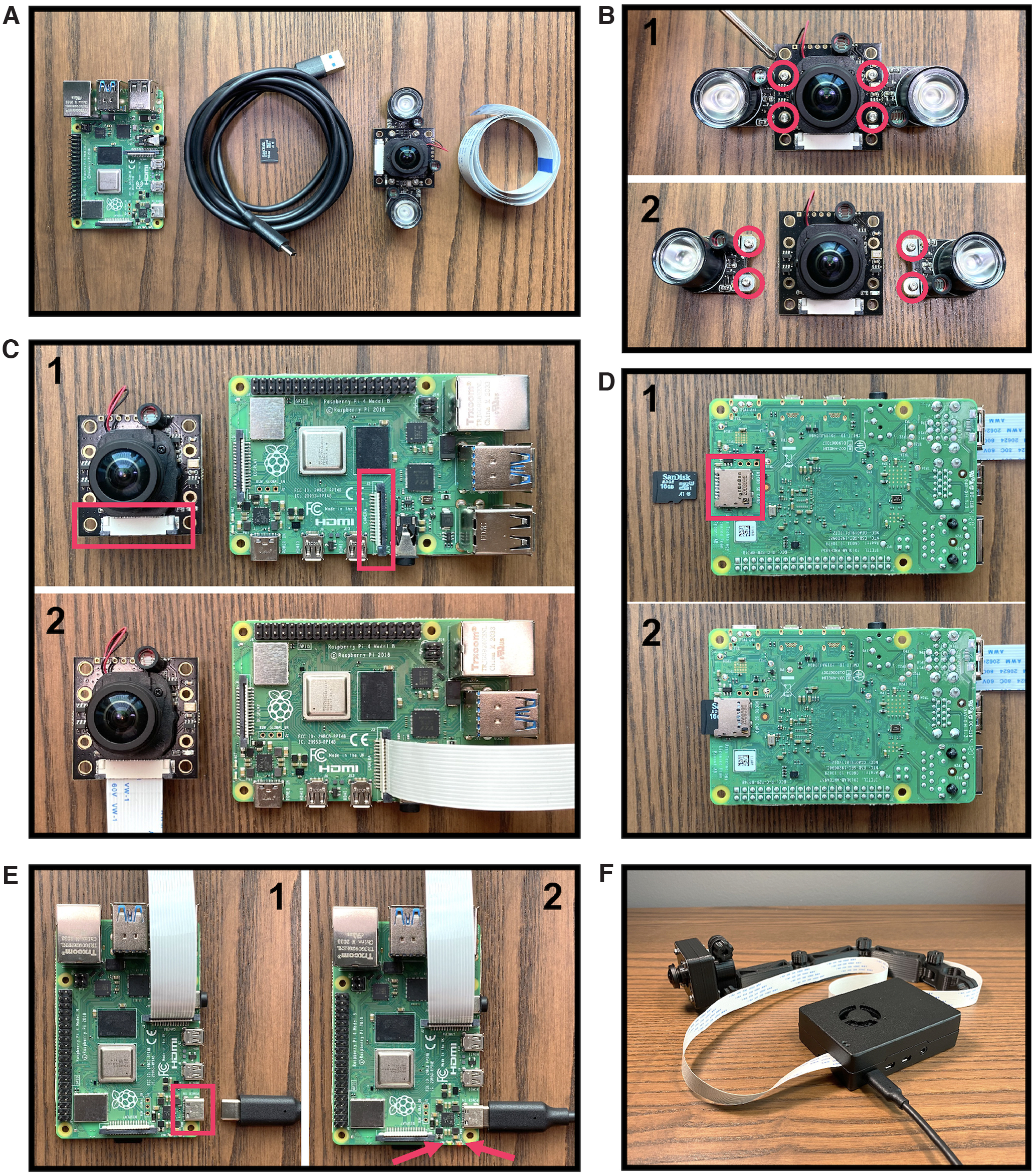
Camera hardware setup. ***A***, Essential components required to build a Pi USB Cam including the main Raspberry Pi board, a USB to USB-C cable, a micro-SD card, the camera module, and a camera ribbon flex cable (listed from left to right). ***B***, Remove the attached IR LEDs from the main camera board by unscrewing the four screws outlined in red. ***C***, Connect the main camera board to the Raspberry Pi 4B board via a camera ribbon flex cable. See Extended Data Figure 2-1 for a labeled diagram of the Pi motherboard. ***D***, Insert the prepared micro-SD card (see Fig. 1 for software setup) into the Raspberry Pi board. ***E***, Power up the Pi USB Cam by connecting it to a computer using the USB cable. ***F***, House the camera in custom 3D-printed case and mount tools for protection and installation in behavioral testing environment.

1. Gather the essential hardware components shown in Figure 2*A* and appropriate housings if desired for the Pi board (Table 1) and camera (Table 2).
  Optional: if you intend to use the camera for low-/no-light recording close to a reflective surface such as Plexiglas, we recommend removing the IR LEDs that come attached to the camera module (Fig. 2*B*) at this stage. Doing so will allow for flexible LED placement thereby enabling the user to avoid flaring artifacts in the captured image (Fig. 4*A*). For more information on how to use and position LEDs independently from the camera refer to “**IR LED Setup**” and see Figure 4.
2. To connect the camera module to the Pi board, first locate the camera ribbon cable ports on the camera module and the Pi board, as indicated in Figure 2*C1*. Gently lift the black plastic clip on the ports. Insert the ribbon cable terminal making sure that the silver leads on the cable face the contacts inside the port. Push down the plastic clip to secure the ribbon cable in place (Fig. 2*C2*).
3. Insert the micro-SD card prepared in *Software Installation* into the micro-SD card slot on the back of the Pi board to finish the setup (Fig. 2*D*).
4. To power up and start using the Pi USB Cam, connect it to a host computer via its USB-C port using a USB cable (Fig. 2*E1*). The red built-in LED should stay lit to indicate that it is receiving adequate power supply (Fig. 2*E2*). The green built-in LED will fast blink three times after booting, which will take around 5 s, to indicate that the Pi USB Cam is ready for use. This LED will remain illuminated when the Pi USB Cam is in active use (Fig. 2*E2*).
5. You can optionally house the Pi USB Cam in our custom 3D-printed camera case for protection and use mount tools for easy installation in a variety of behavioral testing configurations (Figs. 2*F*, 9–11). STL files and print instructions can be found at our Thingiverse page: https://www.thingiverse.com/gloverlab/designs.
  Note: the STL files provided can be readily sliced by popular slicing software and printed with consumer-grade 3D printers or commercial pay-to-print service offered by universities and elsewhere. For an estimated cost of printing a full set of our camera and IR LED holders using either printing options, see Extended Data Table 1-2.

**Table 2:** Custom 3D-printed components

STL file	Picture	STL file	Picture
CAMERA CASE:		IR LED CASE:	
Pi_Cam_Case_Top	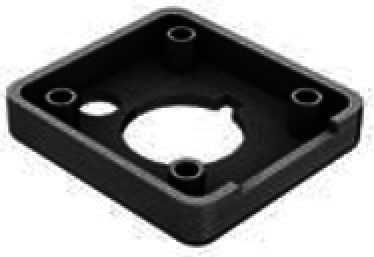	Pi_LED_Case_Top	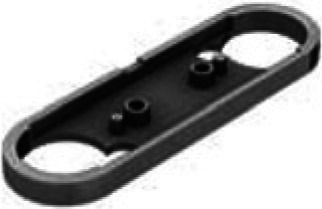
Pi_Cam_Case_Bottom_w_Rod	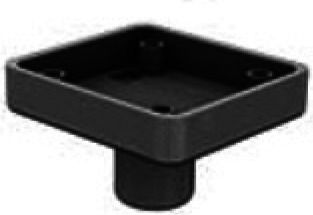	Pi_LED_Case_Bottom_w_Mount	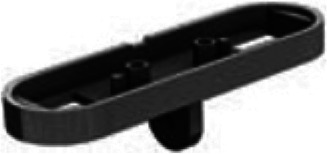
Pi_Cam_w_LED_Case_Top	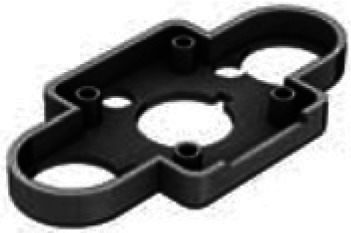	IR LED MOUNT TOOLS:	
Pi_Cam_w_LED_Case_Bottom_w_Rod	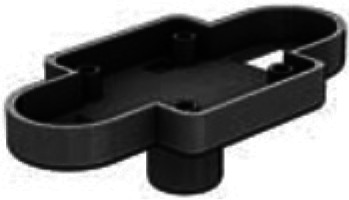	Pi_LED_Arm_5CM_M2F	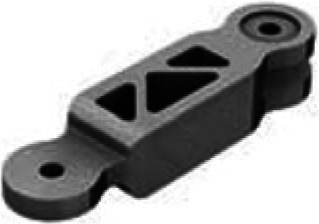
CAMERA MOUNT TOOLS:		Pi_LED_Base	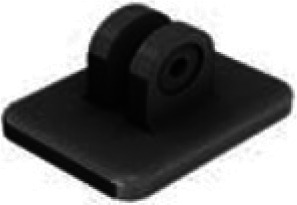
Pi_Cam_Arm_7CM_M2F	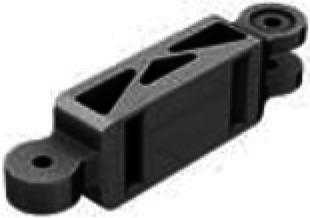	Pi_LED_Ring_180D	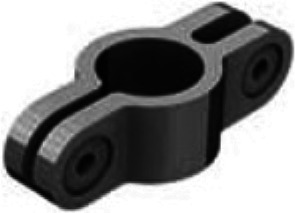
Pi_Cam_Arm_7CM_M2M_90D	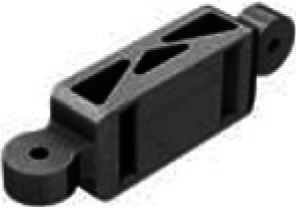	ACCESSORIES:	
Pi_Cam_Arm_7CM_M2M_180D	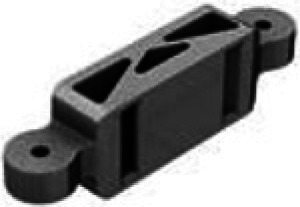	M2.5_Nut_Knob	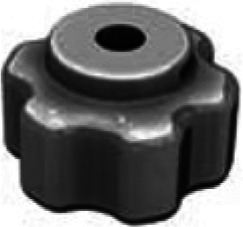
Pi_Cam_Ring_90D	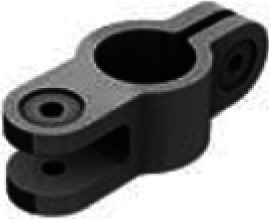	G-Clamp_Clamp	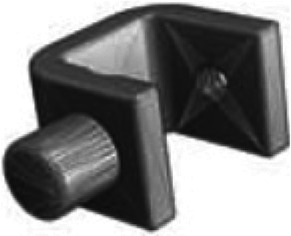
Pi_Cam_Ring_180D	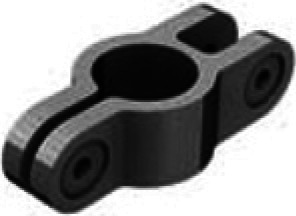	G-Clamp_Screw	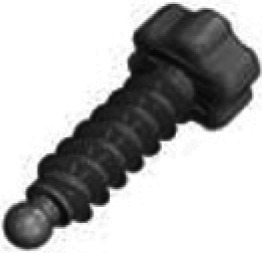
Pi_Cam_Rod_Base	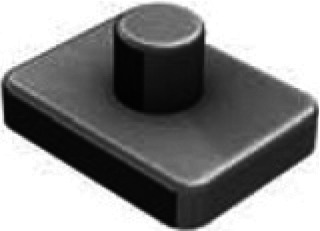	G-Clamp_Press	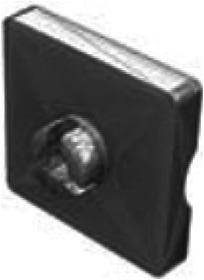

Comprehensive list of all 3D-printed components used in the current build. STL files and print instruction are accessible at https://www.thingiverse.com/gloverlab/designs.

10.1523/ENEURO.0224-22.2022.tab1-1 Extended Data Table 1-1 List of store-bought components including alternative options and accessories. Download Table 1-1, XLS file.

10.1523/ENEURO.0224-22.2022.f2-1 Extended Data Figure 2-1 Diagram of the Raspberry Pi 4B motherboard. Image source: https://github.com/raspberrypi/documentation/blob/develop/documentation/asciidoc/computers/os/using-gpio.adoc. Download Figure 2-1, TIF file.

### Adjusting camera settings

**Figure 3.**
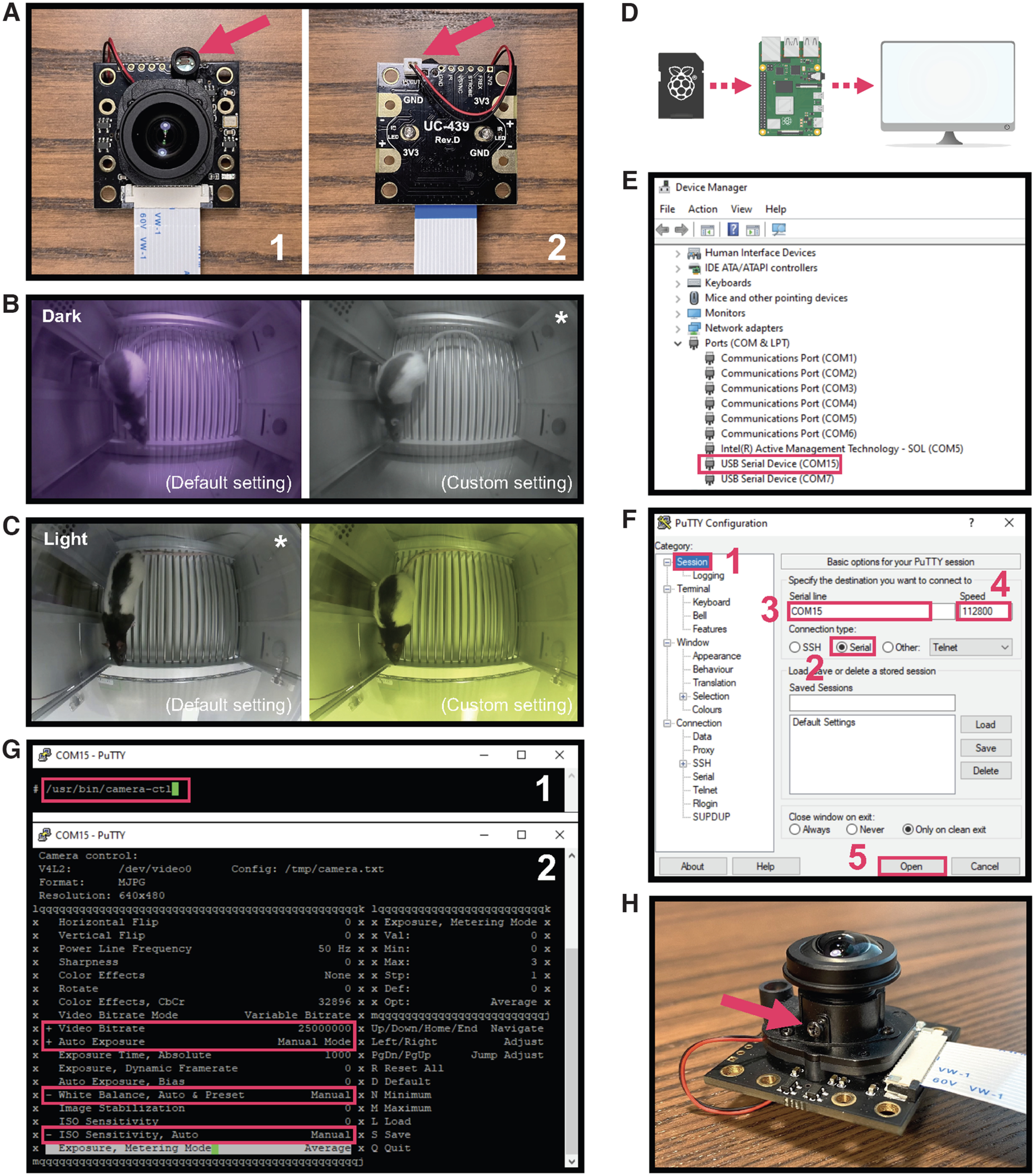
Adjusting camera settings. ***A***, Manually enable IR sensitivity by covering the photoresistor with nontranslucent tape (1) or enable IR correction by unplugging the connector on the back of the camera (2). ***B***, Use of our recommended low-light custom settings (right) eliminates slightly purple hue obtained using the camera’s default settings (left) when recording in total darkness. ***C***, In contrast, the same low-light settings produce an unwanted yellow hue (right) under bright light conditions when default settings (left) produce a more appropriate image. ***D***, To adjust camera settings, connect the Pi USB Cam containing the micro-SD card into a host computer. ***E***, Identify the COM port for the Pi USB Cam in Windows Device Manager. ***F***, Connect to the Pi USB Cam using the open-source software PuTTY. ***G***, Enter the command shown in (1) to access all adjustable camera setting (2). Settings we recommend adjusting for low-light recordings are highlighted in red. ***H***, Adjust the focus by unscrewing the small screw that secures the lens in place (arrow) and twisting the lens in either direction until your desired image quality is achieved. *Recommended settings for each lighting condition.

The Arducam day-night vision camera module that we suggest using here has an on-board photoresistor (Fig. 3*A1*) that senses ambient light intensity. This allows for automatic control of the IR filter to enable IR sensitivity under low-/no-light conditions (Fig. 3*B*) and improve color accuracy under bright light conditions (Fig. 3*C*). However, in scenarios where lighting conditions change dramatically within a single recording or approach the ambient light threshold, one might consider manually disabling the motorized IR filter to prevent automatic IR filter shuttering and keep it in either a permanent ON or OFF state throughout the entire recording session. Covering the photoresistor with nontranslucent tape will prevent ambient light from reaching the sensor thereby enabling IR sensitivity by permanently turning the IR filter OFF (Fig. 3*A1*). Conversely, to manually enable IR correction, the IR filter can permanently be switched ON by unplugging the motorized IR filter connector from the back of the camera board (Fig. 3*A2*).

Camera software settings such as brightness and white balance can also be readily adjusted to suit specific recording needs, such as improving the image color when recording under low-light conditions (Fig. *3B*). Users can access camera setting parameters by interfacing the Pi USB Cam with a host computer. The following protocol (adapted from https://tutorial.cytron.io/2020/12/29/raspberry-pi-zero-usb-webcam/) describes how to access and adjust camera setting parameters on a Windows PC using an open-source software called PuTTY (Fig. 3*G*), which allows the PC to establish a serial connection with Pi USB Cam. The same can be accomplished on a Linux or Mac computer via command line (visit the official debugging guide for more: https://github.com/showmewebcam/showmewebcam).

#### Step-by-step instructions

1. To identify which USB serial communication (COM) port on your host PC the Pi USB Cam is using, open **Device Manager** and locate the COM port number of the camera under **Ports (COM & LPT)** (Fig. 3*E*).
  Note: if you have more than one USB serial device connected to your computer, you can disconnect and then reconnect Pi USB Cam while monitoring hardware changes in **Device Manager** to confirm which COM port is used by which camera.
2. Download, install, and launch PuTTY from https://www.putty.org. To establish a serial connection with the Pi USB Cam, first make sure that **Session** is selected under **Category** (Fig. 3*F1*). Then set **Connection type** to **Serial** (Fig. 3*F2*), enter the COM port number of the camera under **Serial line** (Fig. 3*F3*), and set **Speed** to **112800** (Fig. 3*F4*). Click **Open** to log into the Pi USB Cam (Fig. 3*F5*).
3. Once logged in, type the following command: /usr/bin/camera-ctl and press **Enter** to launch the **camera-ctl** interface and show all adjustable camera setting parameters (Fig. 3*G*). These can be adjusted during live preview to see how any changes affect image quality.
  Optional: we recommend setting **Video Bitrate** to **Maximum (25000000)**, and **Auto Exposure**, **White Balance**, and **ISO Sensitivity Auto** to **Manual** (Fig. 3*G2*), to improve the image coloring under low-light conditions (Fig. 3*B*). We also set **Brightness** to **53** for all low-light recordings used in this paper. For recording under bright-light conditions, consider reverting **White Balance** to the default **Auto** to improve color accuracy (Fig. 3*C*). However, we encourage experimenting with the setting as it likely varies with the recording condition and recording devices.
4. Once finished, press **S** to save the changes in the camera setting parameters, or revert back to default by pressing **D** (resets individual setting to default) or **R** (resets all settings to default). To terminate the serial connection, first press **Q** to quit the **camera-ctl** interface, and then close the PuTTY session window.
5. To adjust the focus on the camera lens, first loosen the screw that secures the lens firmly inside the M12 lens holder, as indicated in Figure 3*H*, then simply turn the lens clockwise or counterclockwise while monitoring the camera preview until the image becomes sharp.

### IR LED setup

**Figure 4.**
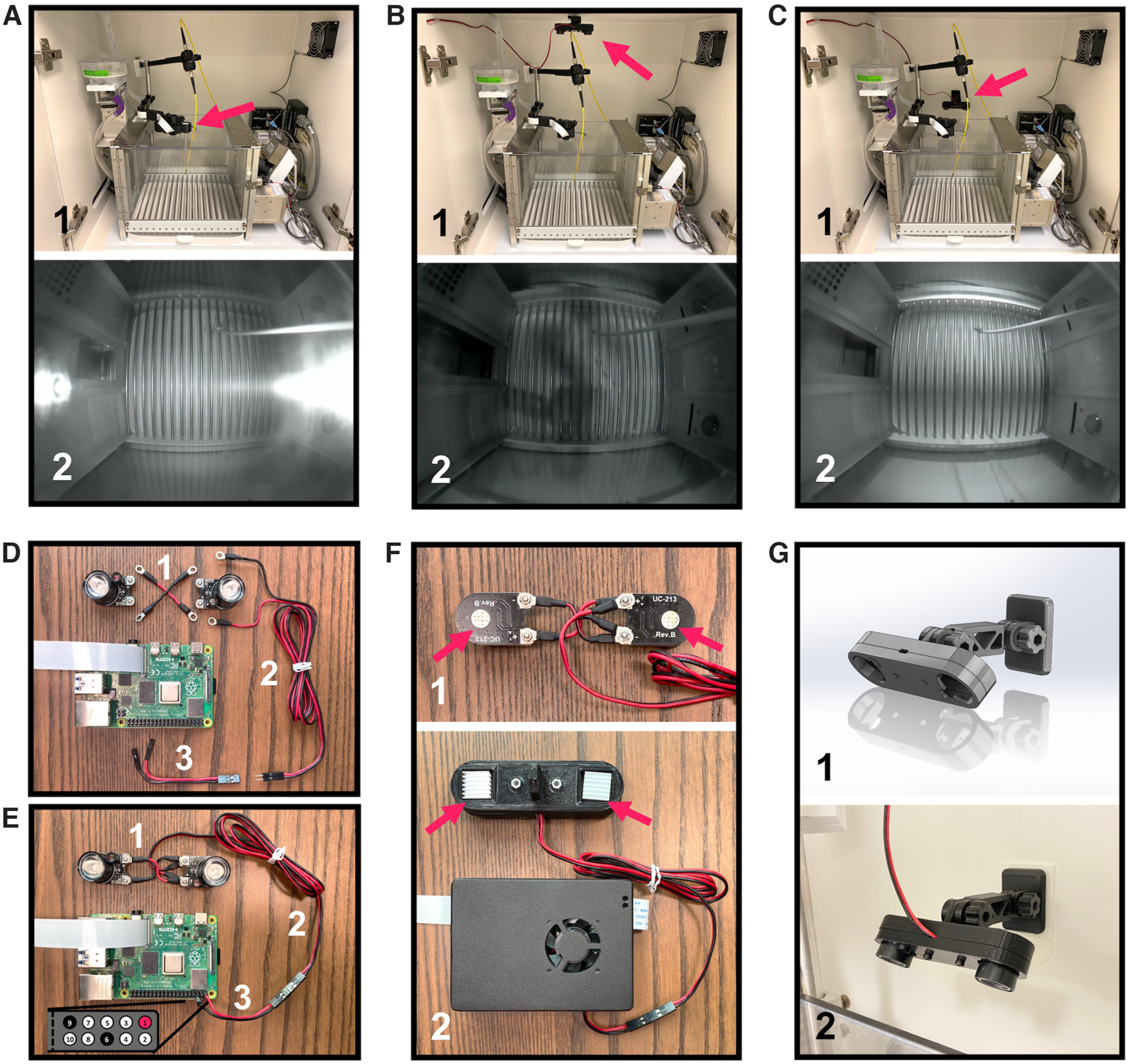
Wiring IR LEDs independent from Pi USB Cam. ***A***, Unwanted flare artifacts are apparent when IR LED boards remain attached to and powered directly by the camera board and the camera is mounted over a reflective surface. Detaching the IR LEDs from the camera board allows for flexible placement in a configuration that avoids these flares as well as shadows cast by other equipment such as what is observed when the LEDs are mounted overhead (***B***) versus away from the reflective surface (***C***). ***D***, Custom-made jumper wires (1–3) necessary to wire the IR LED boards in parallel and power them via a pair of 3v3 power and ground pins on the Pi board. See Extended Data Figures 4-1 and 4-2 for additional wiring instructions. ***E***, Fully connected IR LED unit and Pi board. The locations of 3v3 power pins (highlighted in pink) and ground pins (highlighted in in black) on the 40-pin header are illustrated in the inset. ***F***, Heat sinks should be installed on the back of the LED boards (1) to prevent overheating after housing them in the custom 3D-printed case (2). ***G***, CAD rendering of the IR LED housing and mount parts (1) and a close-up photograph of a fully assembled and mounted IR LED unit (2).

IR-sensitive cameras need to be coupled with adequate IR illumination to produce high-quality images in low-/no-light conditions. The wide-angle day-night vision camera from Arducam that we suggest using in this configuration comes with two 850-nm IR LEDs attached and powered by the camera board (Fig. 2*B*). However, recording through reflective surfaces such as Plexiglas across short object distances (e.g., operant box) caused undesirable flare artifacts in the field of view (Fig. 4*A*). To circumvent this, we removed the LED boards from the main camera board and powered them independently using a pair of 3v3 power and ground pins on the Raspberry Pi board (Extended Data Fig. 2-1). This allowed LED placement independent of the camera enabling even and high-quality illumination of the area of interest (Fig. 4*B*,*C*). This section describes the steps to wire two 850-nm LEDs in parallel using custom-made jumper cables and power them with the Pi board itself (Fig. 4*D–G*).

10.1523/ENEURO.0224-22.2022.f4-1 Extended Data Figure 4-1 Step-by-step instructions for crimping ring terminal connectors. ***A***, Gather all essential parts: (1) electrical wire AWG22, noninsulated ring terminal connector AWG22-16 #4 stud size, heat shrink tubing 1/8”, (2) Astro crimping tool with B-Jaw for noninsulated terminals, and wire stripper. ***B–D***, Strip the wire to expose ∼4 mm of conducting wire, comparable to the length of the wire barrel of the connector. ***D***, Cut one short segment of 1/8” heat shrinking tubing that is long enough to cover the entire wire barrel (≥6 mm) and put it on the wire before crimping. ***E***, Crimp the connector to the bare wire using the first die (DIN 0.5–1.5 mm^2^) on the B-Jaw of the Astro crimping tool. ***F***, Make sure to visually inspect and perform a gentle pull test to confirm the crimp is successful. ***G***, Fully cover the entire wire barrel with heat shrink tubing and use a heat gun to shrink the tubing. Download Figure 4-1, TIF file.

10.1523/ENEURO.0224-22.2022.tab1-2 Extended Data Table 1-2 Pi USB Cam expenses. Laboratories can choose equipment based on budget constraints. Tools and supplies are listed separately because they are only required if users intend to use the IR LEDs independently from the camera as shown in Figure 4 and/or if laboratories are not already stocked with these items. Note that the grand total (*n* = 8) for the upper bound includes the cost of one set of tools and supplies as well as the cost for eight sets of essential equipment. Our university rate for 3D-printing services of $30/kg of PLA plastic was used to calculate the upper bound cost of 3D-printed components, whereas the lower bound was calculated as the expense for only the purchase of plastic for those laboratories that already have a 3D printer. A complete camera (Fig. 2*F*) and IR LED (Fig. 4*G*) holder is estimated to require ∼130 g of PLA plastic based on our recommended print settings. Commercial Webcam = Logitech C930e Webcam; Industrial camera estimates based on quotes from June 2022 from three widely used behavioral neuroscience suppliers. Download Table 1-2, XLS file.

10.1523/ENEURO.0224-22.2022.f4-2 Extended Data Figure 4-2 Step-by-step instructions for crimp pin terminal connectors. ***A***, Gather all essential parts: (1) electrical wire AWG22, male or female pin terminal connector, plastic housing, (2) Astro crimping tool with H-Jaw for open barrel terminals, and wire stripper. ***B***, ***C***, Strip the wire to expose ∼2.5 mm of conducting wire. ***D***, Insert the stripped wire into the pin terminal connector while making sure that the bare wire falls within the wire barrel of the connector and the wire insulation is inside the insulation barrel. Gently bend the insulation barrel around the wire insulation to prevent connector or wire from moving out of place during crimping. ***E***, Crimp the connector to the wire using the middle die (DIN 0.5 mm^2^) on the H-Jaw of the Astro crimping tool. Make sure that the open barrel is facing towards the “nest” of the crimping die and both wire barrel and insulation barrel are positioned in the appropriate spots of the crimping die. ***F***, Visually inspect and perform a gentle pull test to confirm the crimp is successful. ***G***, Insert the crimped connector into a plastic housing. Visually inspection and gentle pull test should be performed to make sure that the connector is securely housed in order to avoid issues with mating pins. Download Figure 4-2, TIF file.

Note: the 3v3 power pin of the Pi board can safely provide up to 500 mA of current (according to: https://pinout.xyz/pinout/3v3_power). Exceeding that limit using high-power LEDs may cause a “brownout” of the Pi and potential safety concerns. Our testing indicated that the two IR LED boards that came with the Arducam day-night vision camera draw ≤200 mA of current from the 3v3 pin when connected in parallel. For higher power LEDs, it is recommended to use either the 5v power pin, which provides a higher current draw of ∼1.5 A (according to: https://pinout.xyz/pinout/5v_power), or an external battery pack or power supply unit. For alternative LED options and related hardware, see Extended Data Table 1-1.

#### Step-by-step instructions

1. Following the step where the LED boards were taken off from the main camera board (Fig. 2*B*), cut three segments of 22-gauge red-black electrical wire (Fig. 4*D*): (1) a short ∼3-cm wire to connect the two LED boards in parallel; (2) a long ∼80-cm wire (adjust to specific installation requirements) for connecting the LED unit to the Pi board located some distance from the camera; (3) a short ∼6-cm wire to connect wire **(2)** to a pair of 3v3 power and ground pins on the Pi board.
2. Split wire **(1)** down the middle into two separate red and black wires. Split one end of wires **(2)** and **(3)** by ∼3 cm and the other end of each ∼1 cm.
3. Crimp the appropriate electrical connectors to the stripped wires as shown in Figure 4*D*.
  - Install two noninsulated ring connectors on either end of wire **(1)**.
  - Install two ring connectors on the end of wire **(2)** separated by ∼3 cm and two male pin connectors housed in one 1x2 plastic housing on the other end.
  - Install two female pin connectors each housed in 1x1 plastic housings on the end of wire **(3)** separated by ∼3 cm and two female pin connectors housed in one 1x2 plastic housing on the other end.
  Note: for detailed instructions on how to crimp ring and pin type connectors, see Extended Data Figures 4-1 and 4-2.
4. Wire the LED boards in parallel as shown in Figure 4*E*,*F1*.
  Note: red wires are used for power (+) and black wires for ground (–). Make sure the polarities of all components align before completing the circuit.
  - Secure the ring connectors on wires **(1)** and **(2)** to the LED boards using the screws that came with camera module.
  - Mate the two female pin connectors on wire **(3)** to any pair of 3v3 power and ground pins on the Pi board 40-pin header (e.g., pins 1 and 6 as shown in Fig. 4*E*).
  - Connect wires **(2)** and **(3)** via their respective pin connectors to complete the circuit.
5. Test functionality by powering up the Pi USB Cam and confirming that the LED unit is illuminated under low-/no-light conditions.
  Install heat sinks on the back of the LED boards to prevent overheating (Fig. 4*F*). Optionally house the fully wired LED unit in the custom 3D-printed case and install the fully assembled unit in any behavioral testing site using the 3D-printed mount tools (Fig. 4*G*).

### Multicamera video acquisition

Pi USB Cam affords a high degree of scalability that is often desired for high throughput experiments. Use of the freely available, open-source video acquisition software, OBS (Open Broadcaster Software) Studio enables preview of live video feeds on screen for real-time behavioral monitoring, live-stream video feeds over Internet, and simultaneous video acquisition from multiple camera sources into separate local files for offline behavioral analysis. OBS Studio is compatible with all three major operating systems; however, we recommend using a Windows PC as the host computer because of added functionalities including video configuration settings that are not available on other platforms. Scalability is limited only by the hardware of the host computer (e.g., number of USB controllers/ports, sufficient CPU, etc.). We recommend using a Windows desktop that allows for add-on USB PCIe expansion cards to provide additional USB controllers and greater bandwidth for multicamera recordings.

The following protocol describes how to set up OBS Studio on a Windows desktop for multi-Pi USB Cam video recordings. For streaming over network, adding audio recording, and many more applications, users can visit the OBS Wiki at https://obsproject.com/wiki/. Note that unless otherwise stated all videos and snapshots of videos included in figures were acquired at 480 p and 30 FPS using OBS Studio.

#### Step-by-step instructions

**Figure 5.**
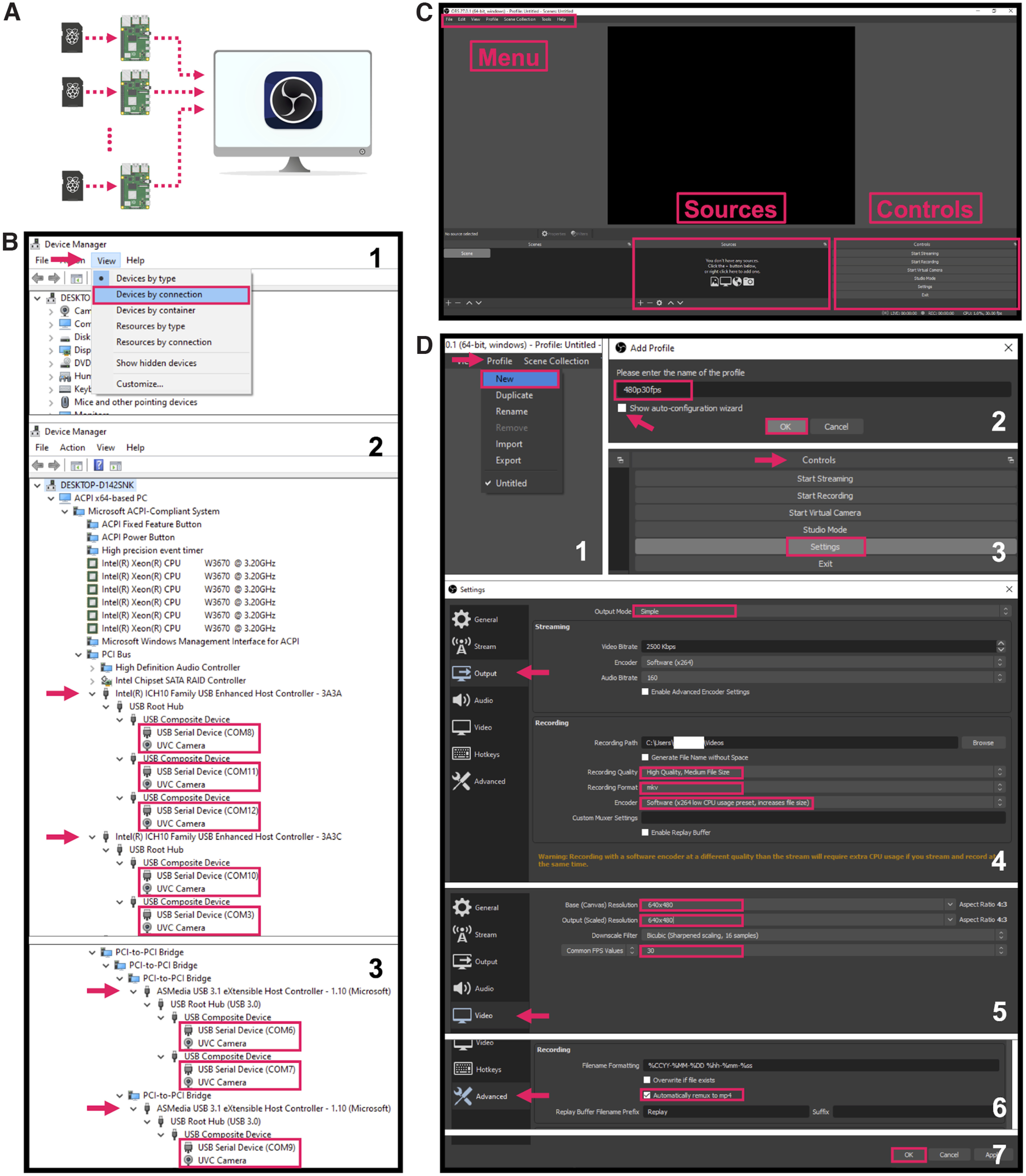
Set up for multicamera recordings. ***A***, The following steps should be done in OBS Studio from a host computer connected to one or more Pi USB Cams. ***B***, Verify in the Windows Device Manager that no more than three cameras are connected to each USB controller. ***C***, Main OBS Studio interface. Menu, Sources, and Controls panels are highlighted. Users can generate a “Profile” from the drop-down menu (***D1***, ***D2***) and use the “Controls” panel to access various recording settings (***D3–D7***). Image source: https://github.com/obsproject/obs-studio/blob/master/UI/forms/images/obs_256x256.png.

**Figure 6.**
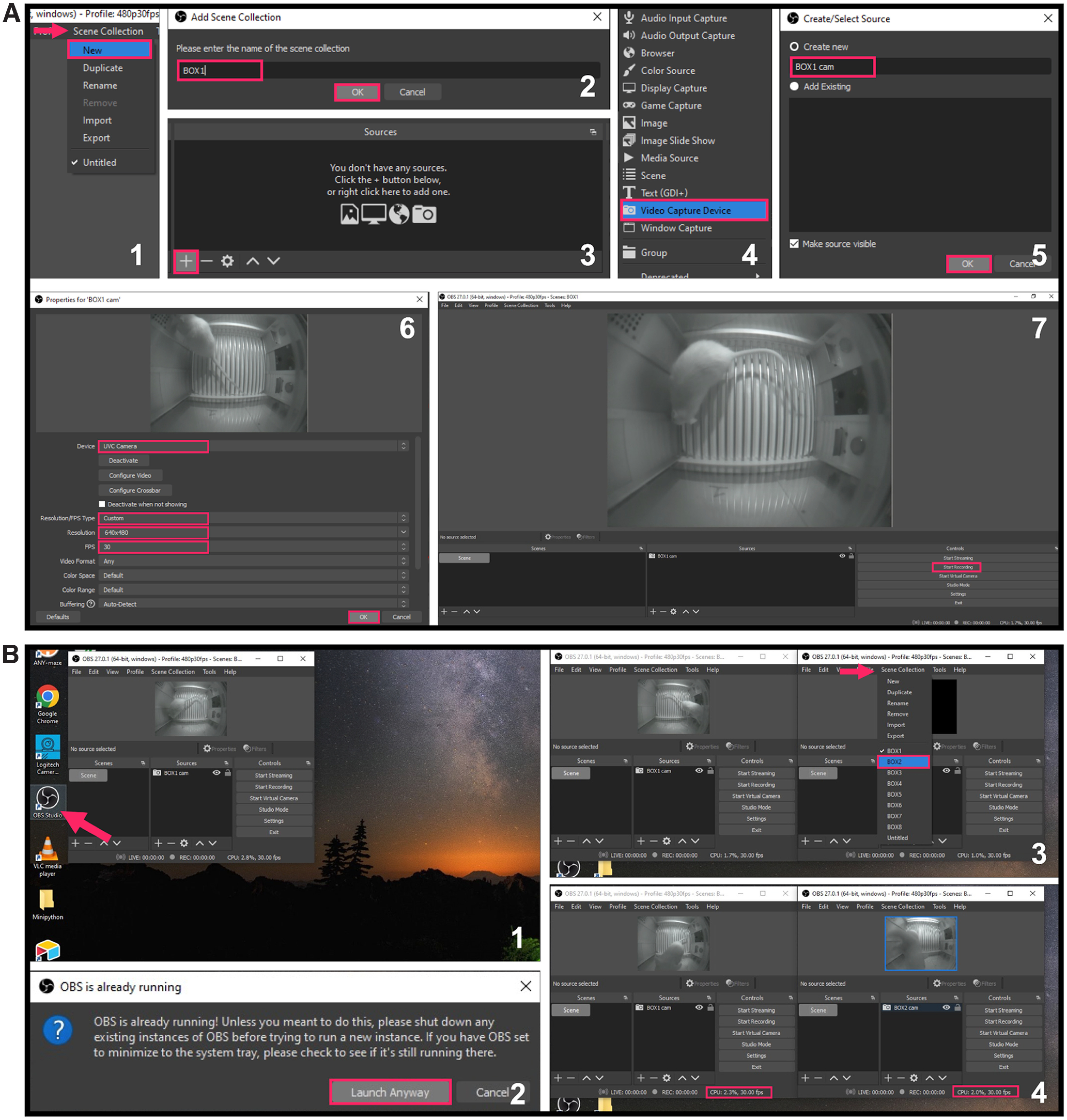
Recommended configuration for separate recordings from multiple video sources. For each Pi USB Cam, create a “Scene Collection” from the drop-down menu (***A1***, ***A2***) and add the camera as a video source using the “Sources” panel (***A3–A7***). ***B***, Launch additional instances of OBS Studio to record from multiple camera sources. See Extended Data Figure 6-1 for a detailed explanation of OBS Studio configurations.

1. Connect one or more Pi USB Cams to a host Windows desktop that is optionally installed with USB PCIe expansion cards for added USB bandwidth (Fig. 5*A*).
2. To make sure that your multicamera configuration will not exceed the USB bandwidth during recording, open **Device Manager**, click **View** followed by **Devices by connection**, and confirm that no more than three Pi USB Cams are connected to each **Host Controller** (Fig. 5*B*).
3. Download, install, and launch the latest release of OBS Studio from: https://obsproject.com (Fig. 5*C*).
  Note: the default user interface can be customized under the **View** menu to hide unnecessary features such as the **Audio Mixer** and **Scene Transitions**.
4. Users can create one or more “profiles” that can save any set of customized recording settings. To do this, go to the **Profile** menu, and click **New** (Fig. 5*D1*). In the pop-up window, enter a name for the new profile (e.g., “480p30fps” to reflect the recording resolution and frame rate that will be updated in step **5**), uncheck the auto-configuration wizard, and click **OK** to finish creating the new profile (Fig. 5*D2*). Once a profile is selected, any changes in recording settings will be automatically saved under that profile.
  Note: one convenient way to use the “profile” feature is to create one for each experimental protocol that specifies its own recording needs and settings. Saved settings for each specific protocol can be quickly applied before each recording session by simply selecting the appropriate profile.
5. To customize recording settings, click **Settings** on the **Controls** panel (Fig. 5*D3*) to open the setting window. Figure 5*D4–D6* highlight several changes to the OBS Studio default settings that we recommend for behavior recordings. Click **OK** to save any changes to the recording settings and to close the pop-up window (Fig. 5*D7*).
6. In steps 6–9, we provide options to preview and record video from multiple video sources. OBS Studio refers to content being broadcast at any given time as a “scene,” whereas a configuration of scenes and their respective video sources is referred to as a “scene collection.” Users may want to select multiple scenes and/or scene collections based on their video needs. For example, multiple video sources can be accommodated within a single scene. This can allow for preview of multiple video sources in tandem. Importantly, recordings in this configuration will include video from each source tiled within a single file (Extended Data Fig. 6-1*A*). Video from multiple sources can also be previewed across separate scenes within a single scene collection. However, OBS Studio is only able to record from one scene at any given time in a multiple scene configuration like this (Extended Data Fig. 6-1*B*). In instances when users want to record video from multiple sources in tandem into separate files, we recommend running multiple scene collections each with a single scene and video source in separate instances of OBS Studio (Extended Data Fig. 6-1*C*). To create one or more “scene collections” that can save any set of “scenes” and “video sources,” go to the **Scene Collection** menu, and click **New** (Fig. 6*A1*). In the pop-up window, enter a name unique to your testing apparatus (e.g., “BOX1” for operant box 1) and click **OK** to finish creating the new scene collection (Fig. 6*A2*).
7. To add a video source to a scene collection, click on the **+** button on the **Sources** panel (Fig. 6*A3*), and select **Video Capture Device** (Fig. 6*A4*). In the pop-up window, enter a name (e.g., “BOX1 cam” for operant box 1 camera) for the new video source, and then click **OK** (Fig. 6*A5*). Once the “properties” window shows up, select the right camera source under **Device**, make additional changes to the device settings, and click **OK** to finish setting up the new video source (Fig. 6*A6*).
8. Click **Start Recording** on the **Controls** panel to start recording and **Stop Recording** to stop (Fig. 6*A7*).
9. For multicamera recording in high throughput experiments (e.g., involving multiple subjects in separate testing sites), repeat steps 6–7 to create one “scene collection” for each camera. Then, launch one more instance of OBS Studio for each additional camera by double-clicking the desktop shortcut (Fig. 6*B1*) followed by clicking **Launch Anyway** on the pop-up warning (Fig. 6*B2*). In each new instance, make sure to select the “scene collection” that contains the correct camera source for the recording site of interest (Fig. 6*B3*). To confirm that the recording will not overload the computer CPU, monitor the CPU usage of each individual instance of OBS Studio provided in the lower right corner of the OBS interface (Fig. 6*B4*) or in **Windows Task Manager**.

10.1523/ENEURO.0224-22.2022.f6-1 Extended Data Figure 6-1 Schematics showing several options to preview and/or record from multiple video sources. ***A***, One scene with multiple video sources operating within one instance of OBS Studio recorded into a single video file. ***B***, Multiple scenes each with their own video source operating within one instance of OBS Studio. Recording produces video from a single scene/video source. ***C***, Multiple scene collections each with their own video source running in independent instances of OBS Studio produce independent video files. Image source: https://github.com/obsproject/obs-studio/blob/master/UI/forms/images/obs_256x256.png. Download Figure 6-1, TIF file.

### Frame rate analysis

Many neuroscience techniques, such as *in vivo* calcium imaging, combine video recordings with optical signal to correlate behavioral events with neural activity. The potential for dropped or duplicate frames, which we have experienced with commercially available USB webcams, poses nontrivial challenges for offline data analysis in these kinds of experiments. Recordings with dropped frames appear fast forward and have shorter duration during playback by third-party media players, which often assume a nominal frame rate that is higher than the actual frame rate achieved. Recordings with duplicate frames appear choppy, and thus have lower temporal resolution than desired, despite the high frame rate achieved. Both issues result in additional and unnecessary workload during postprocessing to accurately align behavioral and neural data. To evaluate the frame rate performance of Pi USB Cam, videos of freely behaving adult male Long–Evans and Sprague Dawley rats were acquired from inside separate operant boxes (MED Associates) using a Pi USB Cam and a generic commercial webcam (Logitech C930e webcam). Camera performance was assessed under both red and white house light illumination.

For dropped frame analysis, Synapse (Tucker-Davis Technologies TDT), an acquisition platform commonly used in neuroscience research, was used to acquire video recordings from both cameras simultaneously. Five videos of ∼5-min duration each were captured into AVI format in each lighting condition from each camera at the maximum resolution (640 × 480) and frame rate (20FPS) supported by Synapse. For each recording, the total number of frames and timestamps of each frame were read from Synapse data block using MATLAB TDTbin2mat function (https://www.tdt.com/docs/sdk/offline-data-analysis/offline-data-matlab/overview/). For each video frame, Synapse stores two timestamps: one for the frame onset and one for offset. The onset timestamp of the very last frame was taken as the recording length, as the offset timestamp of the last frame was stored as *inf* instead of a real number. The true frame rate achieved was calculated using the following equation:

$$true\;FPS=\frac{total\;frames}{recording\;length(s)}.$$

The total number of dropped frames was calculated as:

total dropped frames=target FPS×recording length(s)−1−total frames.

The number of dropped frames per minute was calculated as:

$$dropped\;frames\;per\;min=\frac{total\;dropped\;frames}{recording\;length(s)}\times 60.$$

The video file length when played in third-party media players was calculated as:

$$video\;file\;length(s)=\frac{total\;frames}{target\;FPS}.$$

For duplicate frame analysis, OBS Studio was used to acquire five videos of ∼5-min duration each into mp4 format in each lighting condition from each camera at 640 × 480 resolution and 30 FPS. Visual inspection of videos from each camera made clear that those acquired with the commercial webcam contained abundant duplicate frames, whereas those acquired with Pi USB Cam were essentially devoid of duplicate frames. To quantify this, a custom MATLAB script was used to extract relevant information from each recording, including the recording length and total number of frames, and to identify duplicate frames. Color video files were first converted to grayscale and the frame-by-frame difference in grayscale intensity was calculated for each pixel. Plotting the frequency distribution of maximum change in pixel intensity revealed a bimodal distribution apparent only in videos acquired with the commercial webcam. Using the local minimum as a guide, we identified a threshold for maximum pixel intensity change of 8 for videos acquired under red house light illumination and 10 for videos acquired under white house light illumination. Frames that contain a maximum pixel intensity change below or equal to these thresholds were algorithmically identified as duplicates, which matched the duplicates identified manually in videos acquired with the commercial webcam. The true frame rate of each video, which discounts the duplicate frames and more accurately reflects the temporal resolution, was calculated using the equation:

$$true\;FPS=\frac{total\;frames-total\;duplicate\;frames}{recording\;length(s)}.$$

The number of duplicate frames per second was calculated as:

$$duplicate\;frames\;per\;min=\frac{total\;duplicate\;frames}{recording\;length(s)}\times 60.$$

Results were expressed as mean ± SEM.

### Fisheye distortion correction

Fisheye camera lenses, such as the one used in the recommended Arducam day-night vision camera, allow for capture of a large field of view given a limited object distance. However, they often produce images that are radially distorted, which may pose challenges to accurate position tracking. Here, we describe a simple method to digitally correct fisheye image distortion using a free, open-source OBS Studio plugin, the OBS ShaderFilter.

#### Step-by-step instruction

1. On a Windows PC installed with OBS Studio, download the latest release of OBS ShaderFilter plugin from its GitHub page: https://github.com/Oncorporation/obs-shaderfilter (Extended Data Fig. 8-1*A1*,*A2*), unzip the package file, and drag and drop its contents to the OBS program file directory (Extended Data Fig. 8-1*A3*). The default file location is C:\Program Files\obs-studio. Replace duplicate files if necessary (Extended Data Fig. 8-1*A4*).
2. Next, download the entire repository from its GitHub page and unzip (Extended Data Fig. 8-1*B1*). Locate the “fisheye.shader” text file under the obs-shaderfilter-master\data\examples directory, drag and drop to the OBS program file directory at C:\Program Files\obs-studio\data\obs-plugins\obs-shaderfilter\examples to store alongside other shader filters (Extended Data Fig. 8-1*B2*).
3. Launch OBS Studio and add a video source if you have not (Extended Data Fig. 8-1*C1*; Fig. 6*A*). To import prerecorded videos for offline fisheye correction, add a **Media Source** as the video source (Extended Data Fig. 8-1*C2*). To configure a fisheye camera for real-time fisheye correction during recording, add a **Video Capture Device** as the video source (Extended Data Fig. 8-1*C2*).
4. Right click the video source and select the **Filters** option (Extended Data Fig. 8-1*C3*). In the pop-up window, under the **Effect Filters** header, click the **+** button to add a **User-defined shader** (Extended Data Fig. 8-1*C4*), which can then be renamed to “Fisheye correction” for clarification (Extended Data Fig. 8-1*C5*).
  Note: if the **User-defined shader** is missing, repeat steps 1 and 2 to make sure the plugin is correctly installed.
5. Select the option to **Load shader test from file** and click **Browse** to locate and load the “fisheye.shader” text file. Adjust the **power** parameter to enhance or reduce fisheye effect (Extended Data Fig. 8-1*C6*).
  Note: a positive power adds more fisheye radial distortion while a negative power corrects distortion. A power of zero means no change to the original image. See Extended Data Figure 8-2 for example power settings.
6. In the main OBS Interface, press **Start Recording** to record a new video if a camera has been selected as the source or re-record a video with fisheye distortion corrected if a prerecorded video file has been selected as the source (Extended Data Fig. 8-1*C7*).

### Position tracking and locomotor activity measurement

To assess the effects of fisheye distortion and digital correction on locomotor activity measurement, position tracking was performed on videos with low distortion, fisheye distortion, and distortion digitally corrected. Five videos of ∼5-min duration of adult female Long–Evans rats exploring two contextually distinct compartments in a standard conditioned place preference apparatus (MED Associates) were acquired in OBS Studio from two Pi USB Cams in tandem. One camera was equipped with a 70° HFOV low-distortion lens and the other with a 100° HFOV fisheye lens (Arducam Technology Co, Limited; Extended Data Table 1-1). Videos with fisheye distortion were subsequently corrected using the method described above. Position and locomotor measures were tracked offline using ANY-maze software (Stoelting Co). Each measure obtained was averaged across all five videos and compared using a one-way ANOVA.

For real-time position tracking, a Pi USB Cam was directly interfaced with ANY-maze as a USB camera to provide live video feed.

## Results

### Pi USB Cam offers superior video quality and performance

**Figure 7.**
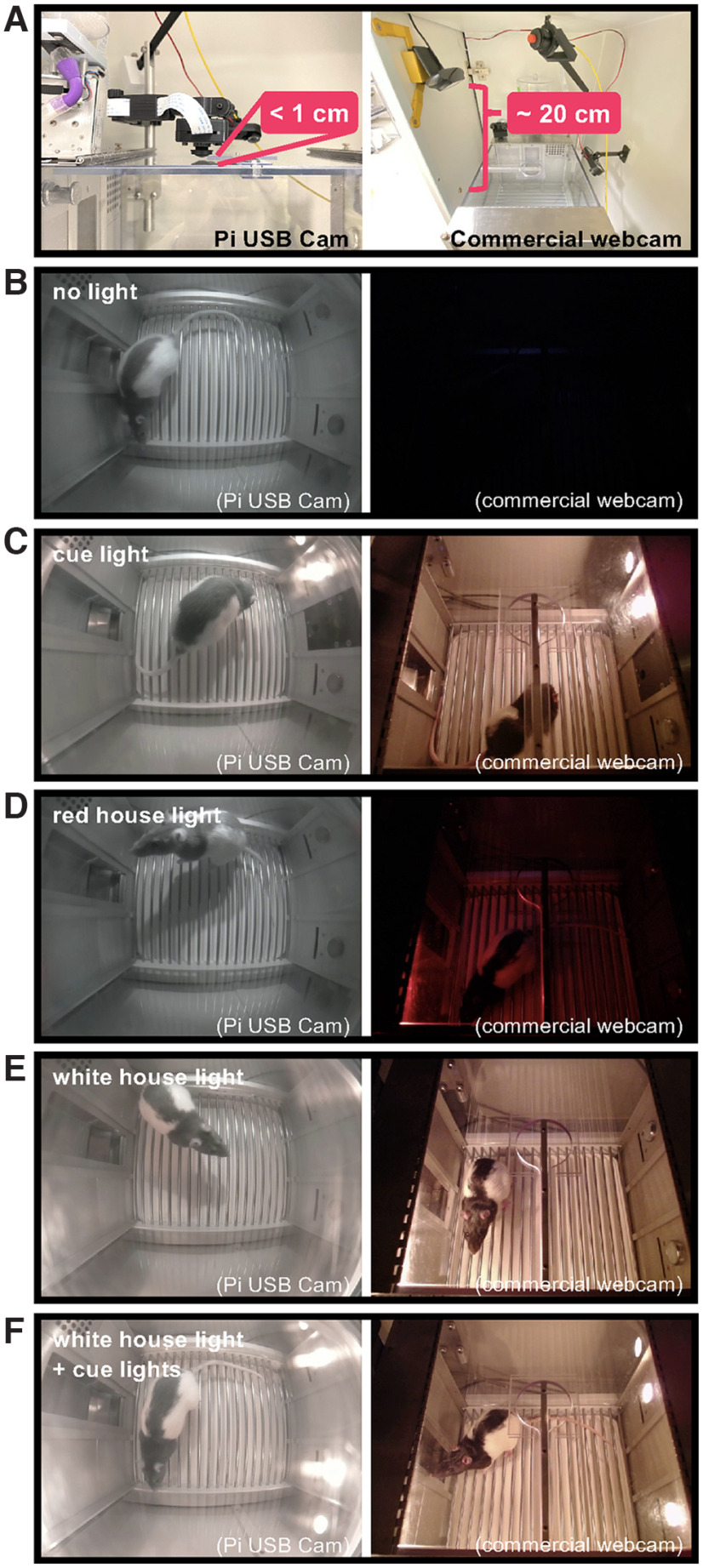
Video quality comparison between Pi USB Cam and commercial webcam. Snapshots of videos acquired using Pi USB Cam (left column) and Logitech C930e webcam (right column) mounted overhead at a distance that accommodated the entire field of view (***A***). Recordings were made under (***B***) no light, (***C***) cue light illumination, (***D***) red light illumination, (***E***) white light illumination, and (***F***) white light illumination + cue lights.

Of significant importance to our research needs was an affordable solution that performed better under low-/no-light conditions within limited object distance compared with commercially available webcams. Pi USB Cam outperformed the Logitech C930e webcam on several measures of significance to behavioral neuroscience research. Monitoring of a standard rat operant testing apparatus with a working area of 11.625” L × 9.78” W × 7.35” H was successfully accomplished with the Pi USB Cam mounted overhead using the out-of-the-box 170° (DFOV) × 140° (HFOV) fisheye lens. Notably, in this orientation, the camera was placed <1 cm above the testing apparatus therefore requiring essentially no additional vertical space for video acquisition (Fig. 7*A*). In contrast, the commercial counterpart, equipped with a 90° DFOV lens, necessitated ∼20 cm of vertical space to capture a similar field of view. Moreover, positioning of the Pi USB Cam allowed for unobstructed access to the roof opening enabling unimpeded movement of tethered animals, whereas angled placement of the Logitech webcam was required to avoid collision with the commutator and tether.

Camera performance was qualitatively assessed under no-visible light condition (Fig. 7*B*), and several standard low-visible light conditions inside an operant box including cue light illumination (Fig. 7*C*), red house light illumination (Fig. 7*D*), white house light illumination (Fig. 7*E*), and white house light + cue light illumination (Fig. 7*F*). With adequate IR illumination, Pi USB Cam provided high image quality regardless of the level of the ambient visible light. In contrast, the Logitech webcam was unable to perform in the no-light condition and image quality was compromised under red house light illumination.

Pi USB Cam also outperformed the commercial webcam in terms of frame rate maintenance. Unlike the Pi USB Cam, the commercial webcam was unable to maintain the set frame rate of 20 FPS when interfacing with Synapse, losing on average 505.60 ± 69.40 frames per min under white house light illumination and 702 ± 0.00 frames per min under red house light illumination (Table 3). This resulted in video files that appear fast forward and shortened when played offline by third-party media players that assume a constant, nominal frame rate of 20 FPS (Movie 1). Pi USB Cam, however, maintained a constant 20 FPS across all trials under both lighting conditions. In contrast to Synapse, OBS Studio was able to achieve the maximum frame rate of 30 FPS with either camera without dropping frames, as determined by the ratio of total number of captured frames to recording length (data not shown). However, videos recorded with the commercial webcam exhibited a high degree of duplicate frames. On average, the webcam contained 1300.20 ± 1.20 duplicate frames per min under white house light illumination and 1303.40 ± 0.24 duplicate frames per min under red house light illumination (Table 4). As a result, these videos appear choppy during playback reflecting reduced temporal resolution despite the high frame rate achieved (Movie 2). Importantly, this analysis cannot distinguish between duplicate frames and instances when the animal does not move between two frames. However, it should be noted that videos acquired with Pi USB Cam were judged to be free of duplicate frames on visual inspection. A similar algorithmic analysis of these videos failed to uncover a clear threshold suggestive of duplicates, as their frequency distributions of maximum change in grayscale pixel intensity appear unimodal as opposed to bimodal. Nevertheless, when the same thresholds used for the commercial webcam were applied to these videos, an average of 466.20 ± 43.24 frames per min were identified for videos under red house light illumination and 310.40 ± 100.74 frames per min for videos under white house light illumination. Not only was the number of identified frames much lower (64–76%) than that observed with the commercial webcam, but this value also varied greatly from video to video. Frames identified as duplicates were also unevenly distributed unlike those observed in videos acquired using the commercial webcam. Combined with visual inspection, these suggest that the algorithmically identified frames in Pi USB Cam videos reflect a lack of animal movement rather than duplicate frames.

**Table 3:** Dropped frame comparison between Pi USB Cam and commercial webcam

Camera	Lighting condition	Recording length (s)	Video file length (s)	Target FPS	True FPS	Total dropped frames	Dropped frames per min
Commercial	Red house light	300.56 ± 6.08	124.77 ± 2.52	20	8.30 ± 0.00	3515.00 ± 71.25	702.00 ± 0.00
Pi USB cam	Red house light	300.60 ± 6.09	300.54 ± 6.09	20	20.00 ± 0.00	0.00 ± 0.00	0.00 ± 0.00
Commercial	White house light	311.05 ± 4.39	179.57 ± 17.43	20	11.57 ± 1.16	2628.60 ± 377.60	505.60 ± 69.40
Pi USB cam	White house light	311.02 ± 4.41	310.96 ± 4.40	20	20.00 ± 0.00	0.00 ± 0.00	0.00 ± 0.00

Five-minute videos (640 × 480 resolution, 20 FPS, avi format) were recorded from each camera via Synapse software for five trials under red and white house light illumination. Results are expressed as mean ± SEM.

**Table 4:** Duplicate frame comparison between Pi USB Cam and commercial webcam

Camera	Lighting condition	Recording length (s)	Target FPS	True FPS	Total duplicate frames	Duplicate frames per minute
Commercial	Red house light	301.29 ± 3.81	30	8.28 ± 0.01	6545.20 ± 83.92	1303.40 ± 0.24
Pi USB cam	Red house light	301.18 ± 4.09	30	30.00 ± 0.00	0.00 ± 0.00	0.00 ± 0.00
Commercial	White house light	300.37 ± 3.77	30	8.33 ± 0.02	6509.00 ± 79.33	1300.20 ± 1.20
Pi USB cam	White house light	300.65 ± 3.47	30	30.00 ± 0.00	0.00 ± 0.00	0.00 ± 0.00

Five-minute videos (640 × 480 resolution, 30 FPS, mp4 format) were recorded from each camera via OBS Studio for five trials under red and white house light illumination. Results are expressed as mean ± SEM.

Movie 1. Comparison of Pi USB Cam and commercial webcam performance using specialized neural recording software for video acquisition. 10.1523/ENEURO.0224-22.2022.video.1

Movie 2. Comparison of Pi USB Cam and commercial webcam performance using OBS Studio. 10.1523/ENEURO.0224-22.2022.video.2

### Pi USB Cam is highly customizable for individual recording needs

The recommended Arducam day-night vision camera can produce high quality video images under both bright and low-/no-light conditions with relative ease (Figs. 7, 9–11; Movies 3, 4) and can also be permanently set to engage or disengage the IR filter (Fig. 3*A*). Moreover, the ability to physically separate the accompanying IR LEDs from the camera body allows for flexibility in illumination options under low-/no-light conditions (Figs. 4, 10*D–F*).

Movie 3. Example video showing versatile implementation of Pi USB Cam for behavioral monitoring. 10.1523/ENEURO.0224-22.2022.video.3

Movie 4. Example video showing versatile implementation of Pi USB Cam for rodent home cage monitoring. 10.1523/ENEURO.0224-22.2022.video.4

**Figure 8.**
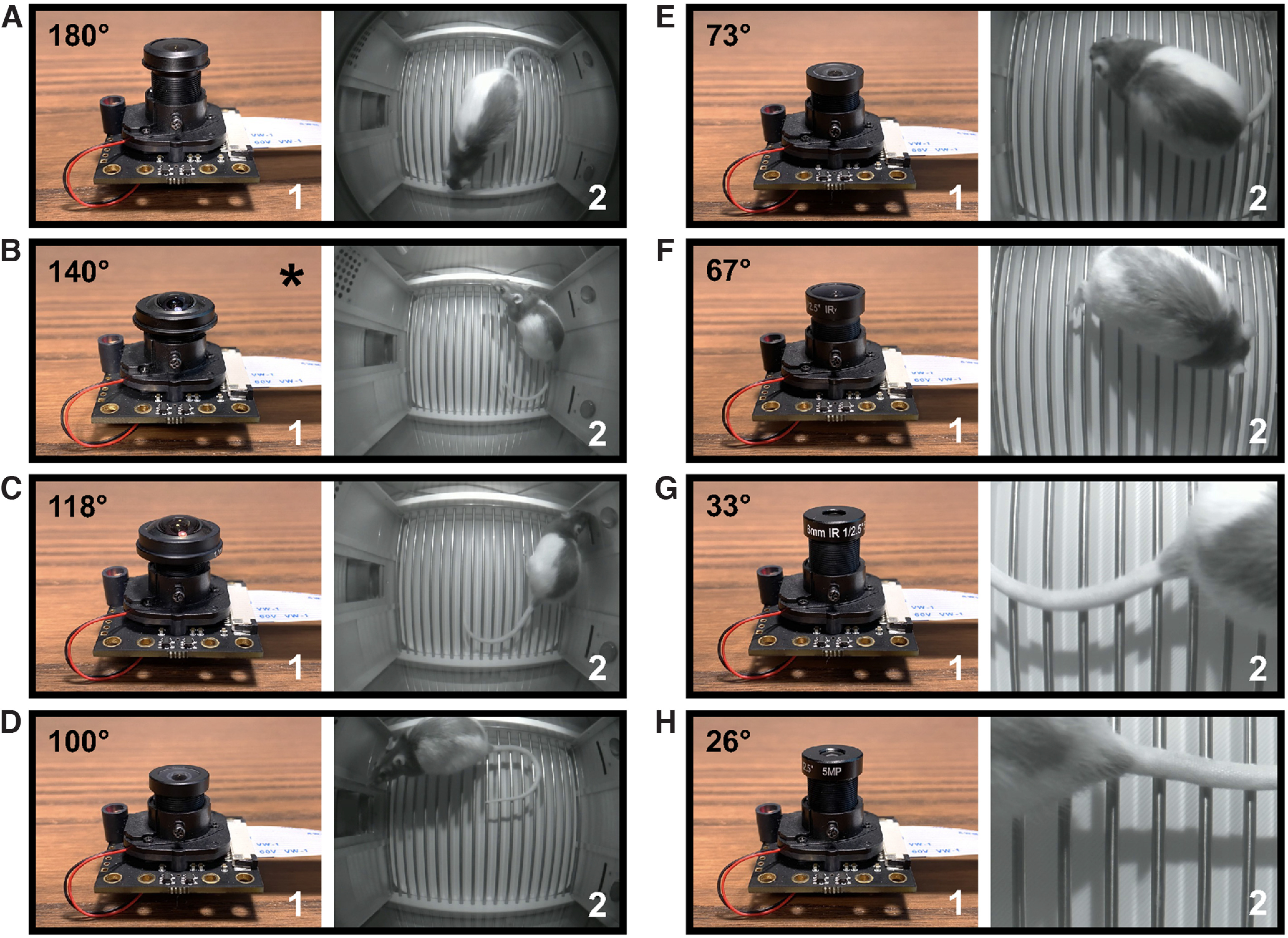
Comparison of different Pi USB Cam-compatible fisheye lenses. Pictures of a Pi USB Cam equipped with various M12 fisheye lenses (1), including 180° (***A***), 140° (***B***), 118° (***C***), 100° (***D***), 73° (***E***), 67° (***F***), 33° (***G***), and 26° (***H***). Corresponding snapshots of videos acquired from a centered position overhead of the arena at a distance of ∼20 cm from the rod floor are depicted for each lens (2). *Indicates the default lens that comes with the Arducam day-night vision camera. See accompanying extended figures for digital fisheye distortion correction (Extended Data Fig. 8-1), comparison of lenses at different object distances (Extended Data Fig. 8-2), and position tracking comparisons under distorted and low-distortion video acquisition settings (Extended Data Fig. 8-3).

We also benchmarked several M12 sized lenses compatible with the Arducam day-night vision camera (see list in Extended Data Table 1-1), each of which is associated with a different field of view and accompanying degree of image distortion. Our research needs necessitate positioning of the Pi USB Cam off-center from the testing arena to accommodate tether access through the roof opening. However, this orientation has the potential to result in uneven image distortion across the field of view as distortion tends to be minimal at the center of the fisheye field of view and progressively enlarged toward the radial edge of the image (Clemensson et al., 2020). Therefore, comparison of different lenses was performed with the camera placed directly above the center of the arena at the level of the Plexiglas roof ∼20 cm above the grid floor measuring 11.5” L × 10.25” D × 1.75” H. As shown in Figure 8, we found that the default lens (140° HFOV) struck the best balance between an adequate field of view to capture the majority of the area of interest and an acceptable amount of image distortion to enable accurate behavioral tracking. Comparison of the object distance required to obtain a similar field of view across different M12 fisheye lenses is provided in Extended Data Figure 8-2.

10.1523/ENEURO.0224-22.2022.f8-1 Extended Data Figure 8-1 Digital correction of fisheye distortion. ***A***, Download the OBS ShaderFilter plugin from its GitHub page and install on a Windows PC equipped with OBS Studio. ***B***, Download the “fisheye.shader” text file located in its GitHub repository. In the main OBS interface, add a prerecorded video as a “Media source” for offline fisheye correction or a camera as a “Video Capture Device” for real-time fisheye correction (***C1***, ***C2***). Enable the ShaderFilter plugin and fisheye correction for each video source (***C3–C7***). Download Figure 8-1, TIF file.

10.1523/ENEURO.0224-22.2022.f8-2 Extended Data Figure 8-2 Comparison of Pi USB Cam-compatible fisheye lenses in terms of object distance and fisheye correction. Pictures showing the object distance of a Pi USB Cam (1) equipped with various M12 fisheye lenses including 180° (***A***), 140° (***B***), 118° (***C***), 100° (***D***), and 73° (***E***) for overhead viewing in a standard operant box. Snapshots of raw videos acquired from a centered position at the corresponding object distances showing comparable captured fields (2), and the same videos after the fisheye image distortion was digitally corrected using the OBS ShaderFilter plugin (3). Power settings used are indicated on each image. *Indicates the default lens that comes with the Arducam day-night vision camera. Download Figure 8-2, TIF file.

10.1523/ENEURO.0224-22.2022.f8-3 Extended Data Figure 8-3 Effects of fisheye distortion on position tracking and locomotor measures. Snapshots of ANY-maze position tracking (1) performed on a video acquired with a 100° HFOV fisheye lens without distortion correction (***A***), after digital distortion correction (***B***), and from a video acquired in tandem using a 70° HFOV low-distortion lens (***C***). Representative center-point tracking plots (2) and heatmaps (3) from the same video show similar results. ***D***, Effects of fisheye distortion and correction on position tracking accuracy were demonstrated by comparing various locomotor activity measures, including the total distance travelled (1) and average speed (2) during the entire test duration, as well as the total distance travelled (3, 4), average speed (5, 6), and time spent (7, 8) in either side of the testing apparatus. Download Figure 8-3, TIF file.

**Figure 9.**
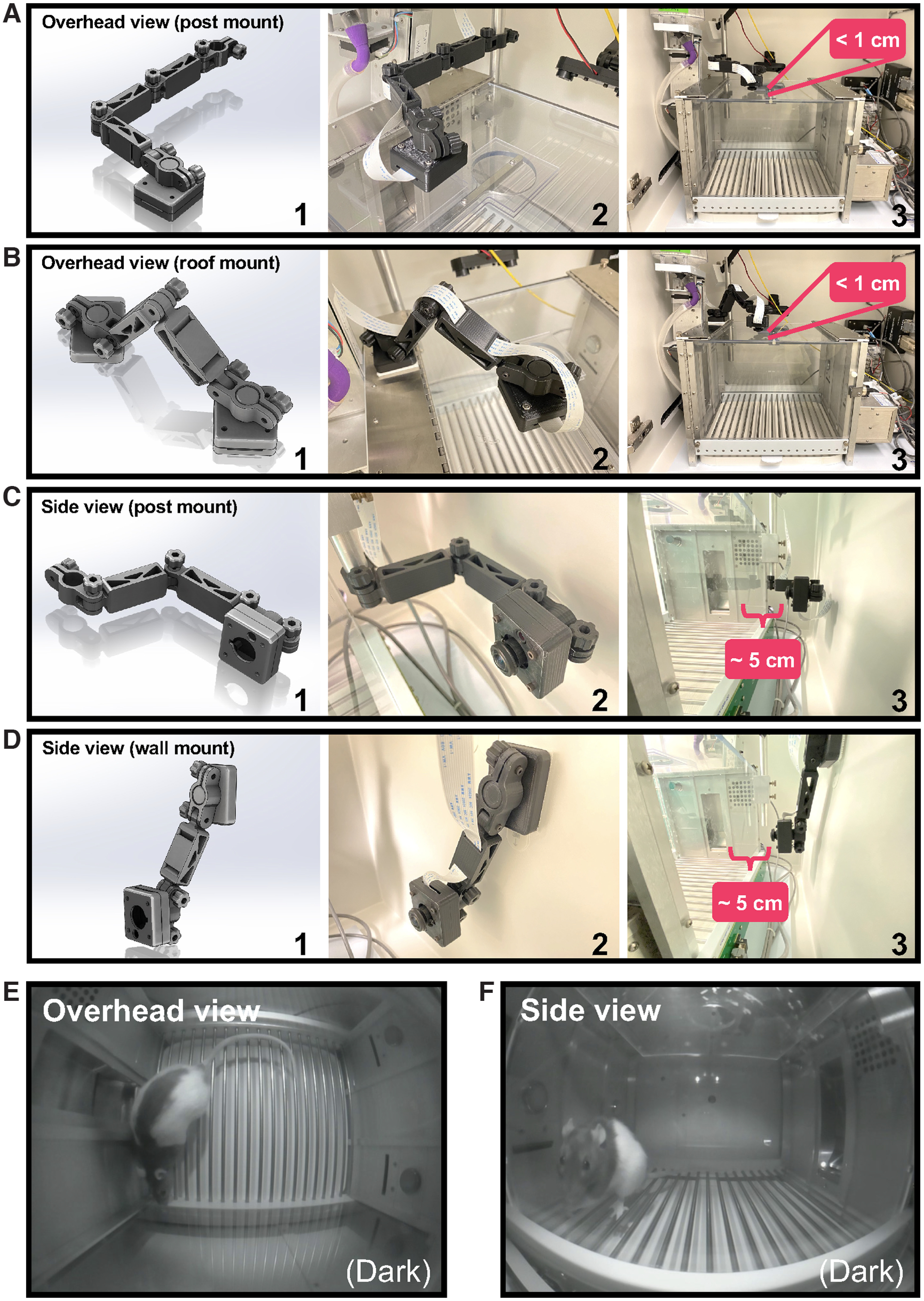
Camera implementation for operant box monitoring. Use of custom 3D-printed components allows for versatile camera installation options including overhead (***A***, ***B***) and side (***C***, ***D***) mounted configurations. For each option, (1) depicts the CAD drawing of 3D-printed components. Close-up photographs of each configuration are provided in (2) with object distance indicated in (3). For overhead viewing, Pi USB Cam can be mounted on a commutator balance arm post (***A***) or directly on the roof panel (***B***). Similar post (***C***) and wall (***D***) mount options are available for side view configuration. Both overhead (***E***) and side view (***F***) configurations allow for full view of the operant arena. Video snapshots acquired under no-light conditions.

Using the default lens, Pi USB Cam was also able to capture a generous field of view when positioned for side viewing (at a distance of ∼5 cm away from the Plexiglas wall; Fig. 9; Movie 3). However, in environments where object distance is not a limiting factor or position accuracy is a primary concern, users can opt for lenses with a narrower field of view and lower image distortion, or lenses designed specifically for minimum distortion (see Extended Data Table 1-1). For example, for overhead monitoring of a standard conditioned place preference testing apparatus with a working area of 32.70” L × 8.25” W × 8” H, we found that a 100° (HFOV) fisheye lens was ideally suited to capture the entire testing arena when the camera was positioned ∼30 cm overhead (Fig. 11; Extended Data Fig. 8-3; Movies 5, 6).

Movie 5. Example video showing position tracking performed on a video acquired with a fisheye lens before and after distortion correction, and a video obtained with a low-distortion lens. 10.1523/ENEURO.0224-22.2022.video.5

Movie 6. Example video showing real-time closed-loop behavioral control by a commonly used video-tracking system combined with Pi USB Cam. 10.1523/ENEURO.0224-22.2022.video.6

### Fisheye distortion correction allows for accurate position tracking and locomotor activity measurement

As an alternative to using low-distortion lenses, which often require larger object distance that is incompatible with many experimental set ups, fisheye distortion can be digitally corrected using the OBS ShaderFilter plugin (Extended Data Figs. 8-1, 8-2, and 8-3; Movie 5) as well as other widely available image processing algorithms. Benchmarked against a low-distortion M12 lens, the fisheye image distortion from a 100° HFOV lens and digital fisheye correction imparted minimal effects on position tracking efficiency (Extended Data Fig. 8-3*A–C*; Movie 5). One-way ANOVA of various locomotor measures revealed a significant between group difference for distance traveled in the white side of the testing apparatus (*F*_(2,4)_ = 6.538, *p* = 0.0462; Extended Data Fig. 8-3*D*). However, *post hoc* comparisons failed to identify a significant difference between precorrection and low-distortion (Dunnett’s test, *p* = 0.0918) or postcorrection and low-distortion (Dunnett’s test, *p* = 0.0783). While no other statistically significant differences were observed, visual inspection of the data makes clear that the difference between precorrection and low-distortion is lessened by digital correction. Therefore, digital fisheye correction is likely to improve tracking accuracy in experiments where absolute, rather than relative, measures are a high priority.

### Custom 3D-printed components afford flexible installation options tailored to individual needs

**Figure 10.**
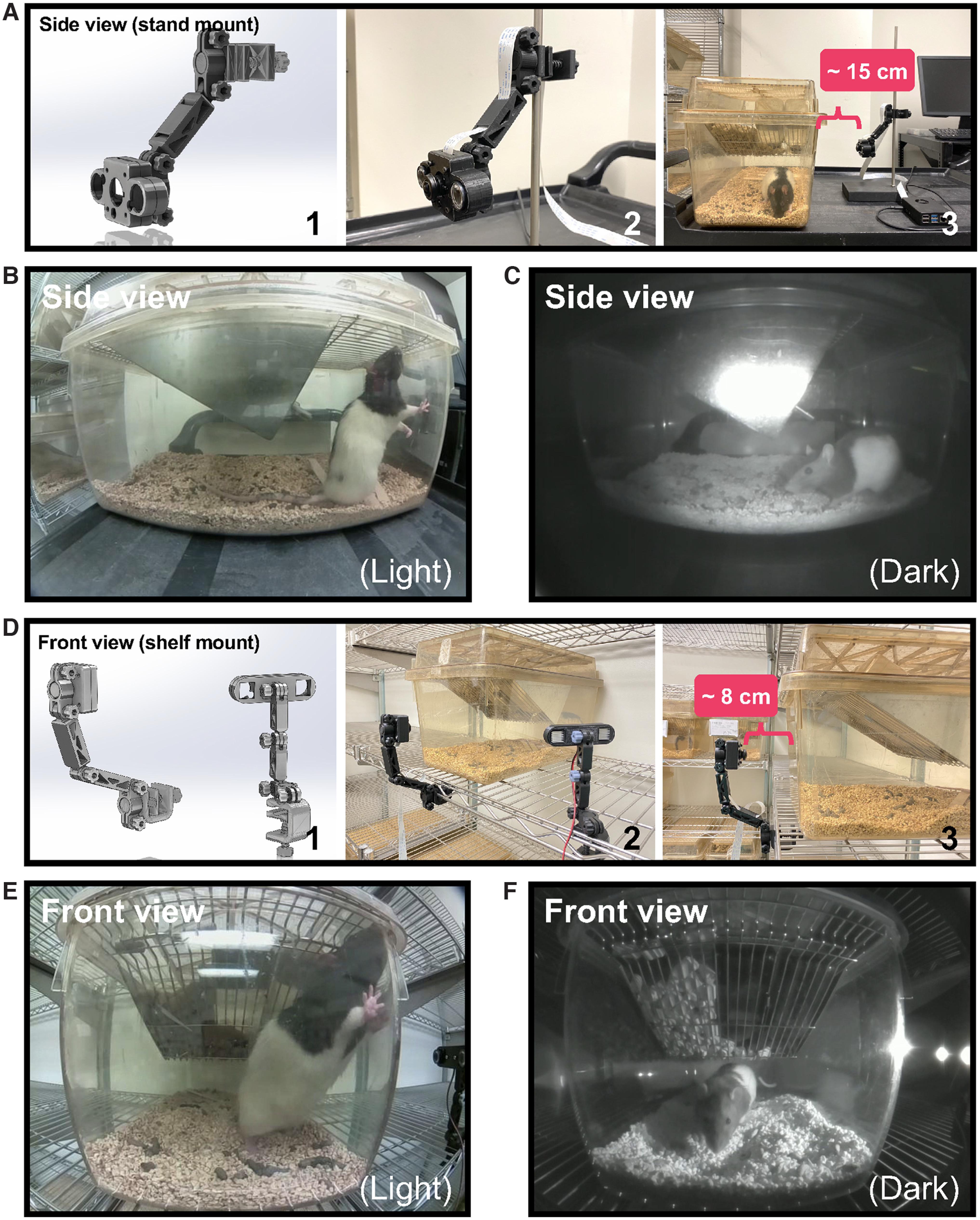
Camera implementation for home cage monitoring. Custom 3D-printed components can also be configured to accommodate home cage recordings in a variety of settings including mount to a ring stand (***A–C***) for recordings performed offsite (e.g., testing room) and on wire shelving (***D–F***) for recordings inside the vivarium. For each option (1) depicts the CAD drawing of 3D-printed components. Close up photographs of each configuration are provided in (2) with object distance indicated in (3). Pi USB Cam is easily mounted to a ring stand (***A***) to accommodate offsite recordings in the home cage shown here in both bright-light (***B***) and no-light (***C***) conditions. Note that in this configuration the IR LEDs are used in the default configuration such that they are attached to and powered directly by the main camera board. Using the custom 3D-printed G clamp, Pi USB Cam (and independent IR LEDs) can also be mounted directly to shelving (***D***) shown here in both bright-light (***E***) and no-light (***F***) conditions.

**Figure 11.**
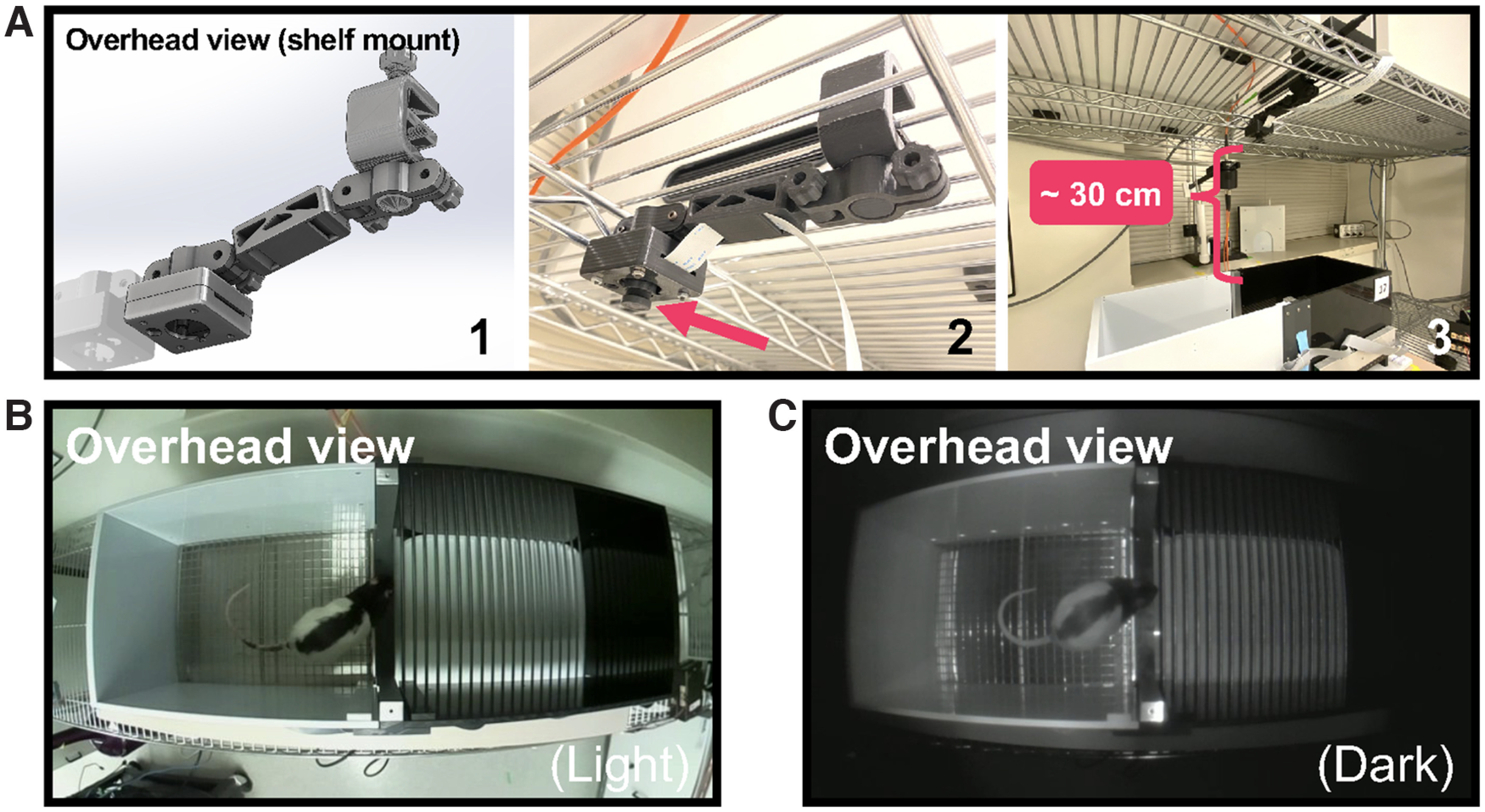
Camera configuration for large apparatus video recordings. Together with custom 3D-printed components, Pi USB Cam can be configured to record from large behavioral testing arenas like the conditioned place preference apparatus shown here. ***A1***, CAD drawing of the 3D-printed components used in this configuration. ***A2***, Close-up photograph of Pi USB Cam mounted overhead on a wire shelf. ***A3***, The entire testing arena is visualized overhead with the camera mounted at a distance of ∼30 cm using a 100° HFOV fisheye lens. Video snapshots acquired with this configuration under bright-light (***B***) and no-light (***C***) conditions.

Our custom 3D-printed components (Table 2) provide added protection and allow Pi USB Cam to be securely mounted on a variety of surfaces or structures. Using these components, Pi USB Cam can be installed on a commutator balance arm post (Fig. 9*A*,*C*), a ring stand (Fig. 10*A*), a wire shelf (Figs. 10*D*, 11*A*), or any flat surface (Fig. 9*B*,*D*). Moreover, the hinged camera mount components provide ample degrees of freedom to finely adjust camera positioning for optimal image acquisition. Thus, use of our custom 3D-printed components ensures Pi USB Cam is readily adaptable to virtually any recording condition. This includes recording within the limited space around a standard operant box inside a sound attenuating cabinet (Fig. 9; Movie 3), and many other common behavioral testing sites such as a home cage environment (Fig. 10; Movie 4) or larger scale testing arenas like a conditioned place preference (CPP) apparatus (Fig. 11).

### Pi USB Cam is highly scalable for multisubject multisite recording needs

**Figure 12.**
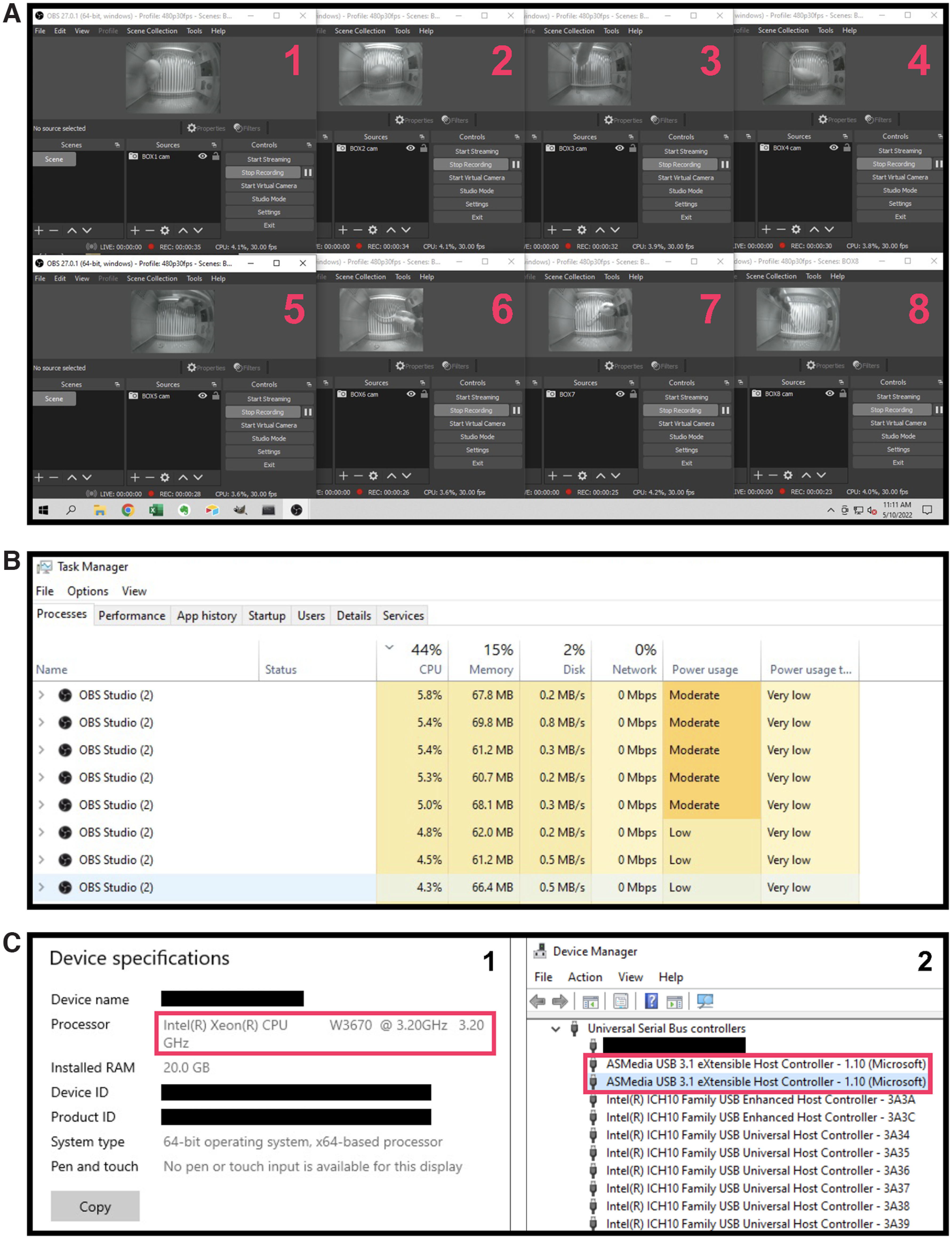
Pi USB Cam scalability is limited only by USB bandwidth and host computer specifications. ***A***, Multisubject multisite recordings are easily achieved using the free and open-source video capture software OBS Studio depicted here with eight independent Pi USB Cams installed in eight separate operant boxes. ***B***, Windows task manager shows the CPU usage of each instance of OBS Studio during multicamera recordings (*n* = 8) using the same video acquisition settings shown in Figure 5*D* and a host computer with the specifications depicted in ***C***, including CPU, memory (1), and add-on USB controllers from a USB PCIe expansion card (2).

Using the free, open-source software, OBS Studio, we were able to successfully video-monitor in real-time eight freely behaving rats in separate operant boxes with eight Pi USB Cams operating simultaneously from a single host computer (Fig. 12*A*). Acquired recordings were subsequently saved into individual video files for further offline analysis. Using our settings (Fig. 5*D*), a 29-min recording produced on average 738.5 MB worth of video file in mkv format (data not shown). At any given moment, each instance of OBS Studio used between 3–6% of the six-core 3.20 GHz CPU on our host computer and the overall CPU usage never exceeded 50% when no other major application was running at the same time (Fig. 12*B*,*C*). Thus, the only major limiting factors to scaling Pi USB Cam are the USB bandwidth and CPU of the host computer.

## Discussion

Pi USB Cam is an affordable, DIY video recording solution that combines simple electronics and 3D printing to enable video monitoring of behavior in diverse recording environments under any lighting condition. Our detailed build instructions are easy to follow and require no specialized expertise. Camera components and accessories are easily sourced from globally accessible vendors. In addition, because DIY components are often discontinued, we provide an extensive list of alternatives that can serve as substitutes if circumstances require. Using this design, we demonstrate its ease of use as a plug-and-play USB camera, with notable superiority over a generic commercial webcam in terms of field of view, IR sensitivity, frame rate, and overall flexibility to meet individual research needs. Lastly, we show that Pi USB Cam, in combination with free, open-source video acquisition software, is easily scalable for multisubject and multisite recordings.

Hardware flexibility is the main advantage of Pi USB Cam over commercially available webcams. Indeed, despite their out-of-the-box user friendliness, the hardware of most commercial webcams is simply not designed to acquire high quality video recordings in confined spaces and/or poor lighting conditions. In contrast, Pi USB Cam can be used with a wide range of camera modules that offer IR sensitivity for low-light recording, IR correction for bright-light recording, fisheye lenses for wide-angle recording, low-distortion lenses for accurate position tracking, etc. Because of its DIY nature, the cost of building Pi USB Cam is comparable to, if not more affordable than, repurposed commercial webcams and a fraction of the cost of video capture systems specialized for neuroscience research (Extended Data Table 1-2). This is particularly important given that camera costs can present significant challenges to scalability and throughput. Augmented by the burgeoning technology of 3D printing, Pi USB Cam can be installed in various commonly used behavioral testing and housing settings without the need for additional modifications to the testing apparatus or additional costly parts.

At the heart of the software design, the *Show-me webcam* firmware enables Raspberry Pi cameras to become an out-of-box, plug-and-play USB camera. In contrast, most, if not all, other DIY solutions require some level of programming proficiency to unlock even the most basic functionalities of the microcomputer and related hardware (Saxena et al., 2018; Singh et al., 2019; Weber and Fisher, 2019; Clemensson et al., 2020; Centanni and Smith, 2021). While DIY solutions tend to turn researchers away because of the steep learning curve associated with such requirements, Pi USB Cam ensures novices need not reinvent the wheel. As a USB camera, Pi USB Cam can be readily integrated into preexisting video recording systems, ranging from the popular open-source recording/streaming app OBS Studio to the professional neural/behavioral recording software Synapse. As such, limited only by the specifications of the host recording computer, Pi USB Cam can be readily scaled to facilitate multisubject, multisite, high-throughput experimental designs. Depending on the recording software of choice, users of Pi USB Cam have the option to watch live video feeds for real-time behavioral monitoring on screen or via network streaming. Users also have the freedom to save video recordings in their preferred file format and location where they can be accessed for offline analysis using sophisticated behavioral tracking algorithms such as ezTrack (Pennington et al., 2019) and DeepLabCut (Mathis et al., 2018). For real-time applications that rely on live video feeds, Pi USB Cam is again able to integrate as a plug-and-play USB camera with powerful, yet widely available neuroscience research applications like ANY-maze, which combine real-time video tracking with various control devices (e.g., optogenetic laser) to achieve closed-loop neural/behavioral manipulation (see Movie 6 for example). Although not tested here, Pi USB Cam is also compatible with recently developed open-source video tracking and control systems, which readily support USB cameras including DeepLabCut-Live (Kane et al., 2020) and DeepLabStream (Schweihoff et al., 2021).

While Pi USB Cam offers an easy-to-use, flexible, and affordable option for behavioral neuroscientists to acquire video recordings, several limitations of our system should be noted. Previous approaches using Raspberry Pi cameras are designed such that the microcomputer performs the heavy lifting of video encoding (Saxena et al., 2018; Singh et al., 2019; Weber and Fisher, 2019; Clemensson et al., 2020; Centanni and Smith, 2021). The advantage of such a design is that it has no inherent limits on scalability other than the number of cameras an individual can purchase. In contrast, Pi USB Cam requires a standalone host computer to perform recordings. In addition to this expense and the footprint associated with a host computer in close proximity to behavioral testing apparatuses, the number of video streams that can be encoded and stored in tandem is limited by the host computer’s processing power and bandwidth. While such a configuration could be problematic for some research needs, we felt that function similar to a commercial webcam makes Pi USB Cam more user friendly and approachable than standalone alternatives. Moreover, connection with a host computer provides significantly greater ease of real-time video monitoring than standalone Raspberry Pi cameras. While *Show-me webcam* is more than adequate for video recording, it does not currently support audio capture. Although this could change in a later release of the firmware, currently video recordings requiring audio measures (e.g., ultrasonic vocalizations, audio cues) necessitate use of a standalone microphone to capture audio independent of video feed, which can then be synchronized online or offline depending on the recording software used. As with any open-source DIY solution, Pi USB Cam software will require regular and timely updates in tandem with upgrades to Raspberry Pi hardware and operating system. However, the well-established open-source community of *Show-me webcam* developers has a strong history of providing regular support and upgrades for the firmware. This is unlike previous niche DIY video solutions, which have often relied on a single individual to perform upgrades and consequently have had difficulty surviving beyond initial hardware and operating system versions (Singh et al., 2019). Finally, our custom 3D-printed case and mount tools require access to a quality 3D printer. However, as the technology of 3D printing matures, small-scale consumer printers are becoming increasingly more affordable, and larger-scale industrial fee-for-service printers are often offered at universities/institutions.

In summary, Pi USB Cam is a highly versatile and affordable DIY video recording solution for real-time behavioral monitoring and offline analysis. Requiring minimum time, expertise, and financial commitment to implement, Pi USB Cam offers behavioral neuroscientists a powerful, yet simple, solution for high quality and high-throughput behavioral data collection. We encourage users to reference the current manuscript when implementing Pi USB Cam in their own experiments.

## Synthesis

Reviewing Editor: Mark Laubach, American University Decisions are customarily a result of the Reviewing Editor and the peer reviewers coming together and discussing their recommendations until a consensus is reached. When revisions are invited, a fact-based synthesis statement explaining their decision and outlining what is needed to prepare a revision will be listed below. The following reviewer(s) agreed to reveal their identity: NONE.

Thank you for submitting your work to eNeuro. Two experts reviewed your paper and their comments are given below in full. Please address all points that were raised in your revised manuscript.

One suggestion of Reviewer 2 was to run an analysis on data collected with your system. Suggestions were to use Optimouse or DeepLabCut. Optimouse depends on having access to Matlab. DeepLabCut requires a good bit of training for a totally new user and access to GPU computing, e.g. using Google Colab. If you have access to these tools or other options (e.g. Ethovision, SCORHE by NIH IDEAS, or the Python based ezTrack), please go ahead and address this concern. If not, please get in touch and we can consult on options. I should note that you might find SLEAP to be a quicker and just as accurate alternative to DeepLabCut.

Reviewer 1

In this manuscript, the authors describe an open source, Raspberry Pi-based USB camera that is designed for easy application of behavioral monitoring. All included instructions are exceptionally clear. This includes not only build instructions, but also step-by-step instructions for using the camera with other open-source programs such as PuTTY for camera setting adjustment, and OBS for multi-camera video acquisition and monitoring. The authors also provide a detailed comparison to commercial USB camera solutions, showing the Pi USB Cam’s superior low light performance and lower susceptibility to dropped frames - a significant issue for time correlated measures of behavior such as in vivo calcium imaging. The provision of alternative sources for the store bought parts is also appreciated.

Major Comments: While the authors do a good job of presenting a method of avoiding some of the glare and shadows by moving the IR lighting source, it would be important to discuss how the recordings might be impacted under low light by cue lights. Currently Figure 7E included both the house light and the cue lights. It would be good to have Figure 7A with a cue light on.

Minor comments:

1. Line 167: Correct “Linus” to Linux.

2. Line 354: TDT link is broken.

3. Line 612: GitHub link is broken.

4. Line 685: GitHub link is broken.

Reviewer 2

The manuscript presents a versatile, affordable and high-throughput DIY video recording solution for behavioral neuroscientists to enable real-time behavioral monitoring and recordings using Pi USB Cam. I commend the authors for their meticulous and clear and instructions. This method is certainly of interest to the broad neuroscience audience interested behavioral measurements as this lowers the threshold for recording behavior of single or multiple animals simultaneously. A big benefit of the proposed method is how it enables home-cage recordings without the need for highly specialized racks which allow for behavioral tracking. This is further enabled by the scalability of this affordable setup. This reviewer can see this method likely being widely adapted by a broad neuroscience audience.

Four major claims of the paper are:

1. The method using Pi USB Cam combines ease of use as a plug-and-play USB camera with flexibility in functions

2. Scalability to facilitate multi-subject, multi-site, high-throughput

3. Improved frame rate performance to commercially available webcams

4. The combines system is considered affordable when compared to readily available systems for behavioral neuroscientists to monitor and record animal behavior

Claims 1, 2 and 4 are well justified and instructions for setting up as well as costs are described highly meticulous and clear to understand.

Claim 3 has been supported by measuring the dropped frames in recorded videos from the Pi USB Cam as well as a very common USB webcam. Surprisingly, this was done in videos recorded in 20 FPS. This is not consistent with the rest of the figures (as stated in line 271-273: “Note that unless otherwise stated all videos and snapshots of videos included in Figures were acquired at 480p and 30 fps using OBS Studio.”), and consists of a significant drop in frame rate which is a very substantial loss when trying to record animal behavior. It would be important for the authors to address this discrepancy, as well as measuring the frame loss at 30 FPS.

Other major points:

It would be of added value to see a simple example of a video which has been ran through a tracking software, for example the open source Optimouse or DeepLabCut. One concern for locomotor activity measurements which was not addressed adequately in the manuscript lays in the strong fisheye effect of the used lens. This effect will cause very strong artifacts in the locomotor measurements based on the location of the animal inside the cage. A suggested method for adjusting the FOV (for example through OBS plugin/filter) would strongly increase the experimental validity of the proposed method.

Line 392/table 3: The results are reported as {plus minus}SEM but number of repetitions is missing. 702{plus minus}0 suggests that number of repetitions might be just a single recording? Please add the number of repetitions and if needed an explanation for how multiple videos of different lengths can all have exactly 702 dropped frames.

Line 422-424: It would be nice to see a comparison of lenses included in figure 8 with those same lenses, but at heights relevant for the lenses. This would be of added benefit for setups where the camera cannot be positioned directly over the arena. It might also help with the fisheye distortion seen in other lenses.

Minor issues:

Line 89: It would be of added benefit to this proposed method if a note could be made of how applicable/easily useable this setup and method is on earlier versions of the Raspberry Pi.

Line 59-60, 489: It is described that previous systems often lack interface with close-loop behavioral control systems. However, while it is true that the current camera can be adapted for such a system, this cannot be done using solely the current method. Other software (such as DeepLabCut) and integration will be needed, but with that those other systems can be made to function with closed-loop as well. Please either remove the statement or expand on it.

## Author Response

We appreciate the reviewers’ enthusiasm for Pi USB Cam and the constructive feedback provided during the peer review of our manuscript (eN-MNT-0224-22). We thank the reviewers for their helpful suggestions and have revised the manuscript accordingly. Below is a point-by-point response to reviewer comments. Changes to the manuscript text are highlighted in blue font.

REVIEWER #1

• Comment 1: While the authors do a good job of presenting a method of avoiding some of the glare and shadows by moving the IR lighting source, it would be important to discuss how the recordings might be impacted under low light by cue lights. Currently Figure 7E included both the house light and the cue lights. It would be good to have Figure 7A with a cue light on.

Response: We appreciate the suggestion and have added panel C to Figure 7 depicting the image quality produced under illumination by a single cue light for both Pi USB Cam and commercial webcam.

• Comment 2: Line 167: Correct “Linus” to Linux. Line 354: TDT link is broken. Line 612:

GitHub link is broken. Line 685: GitHub link is broken.

Response: We apologize for these errors. We have corrected the spelling error and fixed the broken links by redoing the word-to-pdf conversion outside of Word macOS.

REVIEWER #2

• Comment 1: Four major claims of the paper are: 1. The method using Pi USB Cam combines ease of use as a plug-and-play USB camera with flexibility in functions 2. Scalability to facilitate multi-subject, multi-site, high-throughput 3. Improved frame rate performance to commercially available webcams 4. The combined system is considered affordable when compared to readily available systems for behavioral neuroscientists to monitor and record animal behavior Claims 1, 2 and 4 are well justified and instructions for setting up as well as costs are described highly meticulous and clear to understand. Claim 3 has been supported by measuring the dropped frames in recorded videos from the Pi USB Cam as well as a very common USB webcam. Surprisingly, this was done in videos recorded in 20 FPS. This is not consistent with the rest of the figures (as stated in line 271-273: “Note that unless otherwise stated all videos and snapshots of videos included in Figures were acquired at 480p and 30 fps using OBS Studio.”), and consists of a significant drop in frame rate which is a very substantial loss when trying to record animal behavior. It would be important for the authors to address this discrepancy, as well as measuring the frame loss at 30 FPS.

Response: We apologize for the confusion regarding our analysis of dropped frames. To clarify, 20 FPS is the maximum frame rate that the specialized acquisition software, Synapse, supports. We have clarified this in the frame rate analysis section (lines 344- 346). We have also added frame rate analysis for videos recorded at 30 FPS using OBS Studio. As described in lines 359-377 & 463-482, both Pi USB Cam and the commercial webcam were able to successfully capture 30 FPS without dropping frames using OBS Studio. However, many of the frames captured by the commercial webcam appear as duplicates, resulting in a temporal resolution that falls far below the frame rate. We provide a quantitative analysis of these videos in Table 4 and an example recording in Movie 2.

Comment 2: It would be of added value to see a simple example of a video which has been ran through a tracking software, for example the open source Optimouse or DeepLabCut. One concern for locomotor activity measurements which was not addressed adequately in the manuscript lays in the strong fisheye effect of the used lens. This effect will cause very strong artifacts in the locomotor measurements based on the location of the animal inside the cage. A suggested method for adjusting the FOV (for example through OBS plugin/filter) would strongly increase the experimental validity of the proposed method.

Response: We appreciate the reviewer calling attention to the image distortion produced by fisheye lenses and its potential effects on position tracking accuracy. To address this, we now provide instructions for digital correction of fisheye distortion using the OBS ShaderFilter plugin in lines 380-418 and Extended Figure 8-1. We have also added a new figure illustrating the effect of fisheye correction for various lenses (Extended Figure 8-2).

Finally, we benchmarked distortion-uncorrected and -corrected videos against a video obtained with a low-distortion lens and performed quantitative comparison of various locomotor measures using the commonly used video tracking software ANY-maze (lines 420-433 & 514-530, Extended Figure 8-3, Movie 5). Altogether these additions show that while fisheye distortion may contribute to altered accuracy of behavioral tracking data, this can be easily mitigated using the proposed digital correction method.

• Comment 3: Line 392/table 3: The results are reported as {plus minus}SEM but number of repetitions is missing. 702{plus minus}0 suggests that number of repetitions might be just a single recording? Please add the number of repetitions and if needed an explanation for how multiple videos of different lengths can all have exactly 702 dropped frames.

Response: We apologize for the confusion. As explained in line 344, these results are averaged across five recordings. The rate of dropped frame per minute was identical across all five recordings acquired with the commercial webcam under red house light illumination, demonstrating the camera’s highly consistent, though deficient, video acquisition properties and explaining the SEM of zero. For additional clarity we have added a column to Table 3 indicating the total dropped frames +/- SEM. Each video was ∼ 5 mins long and because recordings of different lengths have different numbers of dropped frames the SEM in this column is not zero.

• Comment 4: Line 422-424: It would be nice to see a comparison of lenses included in figure 8 with those same lenses, but at heights relevant for the lenses. This would be of added benefit for setups where the camera cannot be positioned directly over the arena.

It might also help with the fisheye distortion seen in other lenses.

Response: We appreciate the reviewer suggestion and have added Extended Figure 8- 2 which shows the requested comparison.

• Comment 5: Line 89: It would be of added benefit to this proposed method if a note could be made of how applicable/easily useable this setup and method is on earlier versions of the Raspberry Pi.

Response: The show-me webcam firmware currently supports Raspberry Pi 4B, Zero v1.3 (without wifi), and Zero W (with wifi), but not legacy boards that are no longer widely accessible for purchase. We have edited the text in lines 88-89 to clarify this.

• Comment 6: Line 59-60, 489: It is described that previous systems often lack interface with close-loop behavioral control systems. However, while it is true that the current camera can be adapted for such a system, this cannot be done using solely the current method. Other software (such as DeepLabCut) and integration will be needed, but with that those other systems can be made to function with closed-loop as well. Please either remove the statement or expand on it.

Response: We agree with the reviewer that the potential application of Pi USB Cam for close-loop behavioral control warrants more discussion. To address this, we have expanded our discussion on this issue (lines 432-433, lines 597-604) and provided an additional example video (Movie 6) demonstrating real-time position tracking and closed loop optogenetic control by interfacing Pi USB Cam with the commonly used video tracking software ANY-maze. These additions show that Pi USB Cam is easily paired with specialized software programs that accept video capture from USB cameras to facilitate real-time behavioral tracking and/or closed-loop behavioral control without the need for additional modification. It should be noted that DeepLabCut is designed for offline behavioral analysis of pre-recorded videos and therefore cannot support closed-loop behavioral optogenetics methods. However, more recently developed open-source solutions like DeepLabCut-Live (Kane et al. 2020) and DeepLabStream (Schweihoff et al. 2021), which apply AI-based pose estimation to enable real-time video tracking and closed-loop behavioral control using live camera feeds, are becoming increasingly popular. While our laboratory is not currently using any of these applications, they readily support USB cameras and are therefore compatible with Pi USB Cam.
