# Supplementary figures and images for: Pi USB Cam: A Simple and Affordable DIY Solution That Enables High-Quality, High-Throughput Video Capture for Behavioral Neuroscience Research
Source: eNeuro. 2022 Sep 23;9(5):ENEURO.0224-22.2022. doi: 10.1523/ENEURO.0224-22.2022 (PMC9522465; doi:10.1523/ENEURO.0224-22.2022)

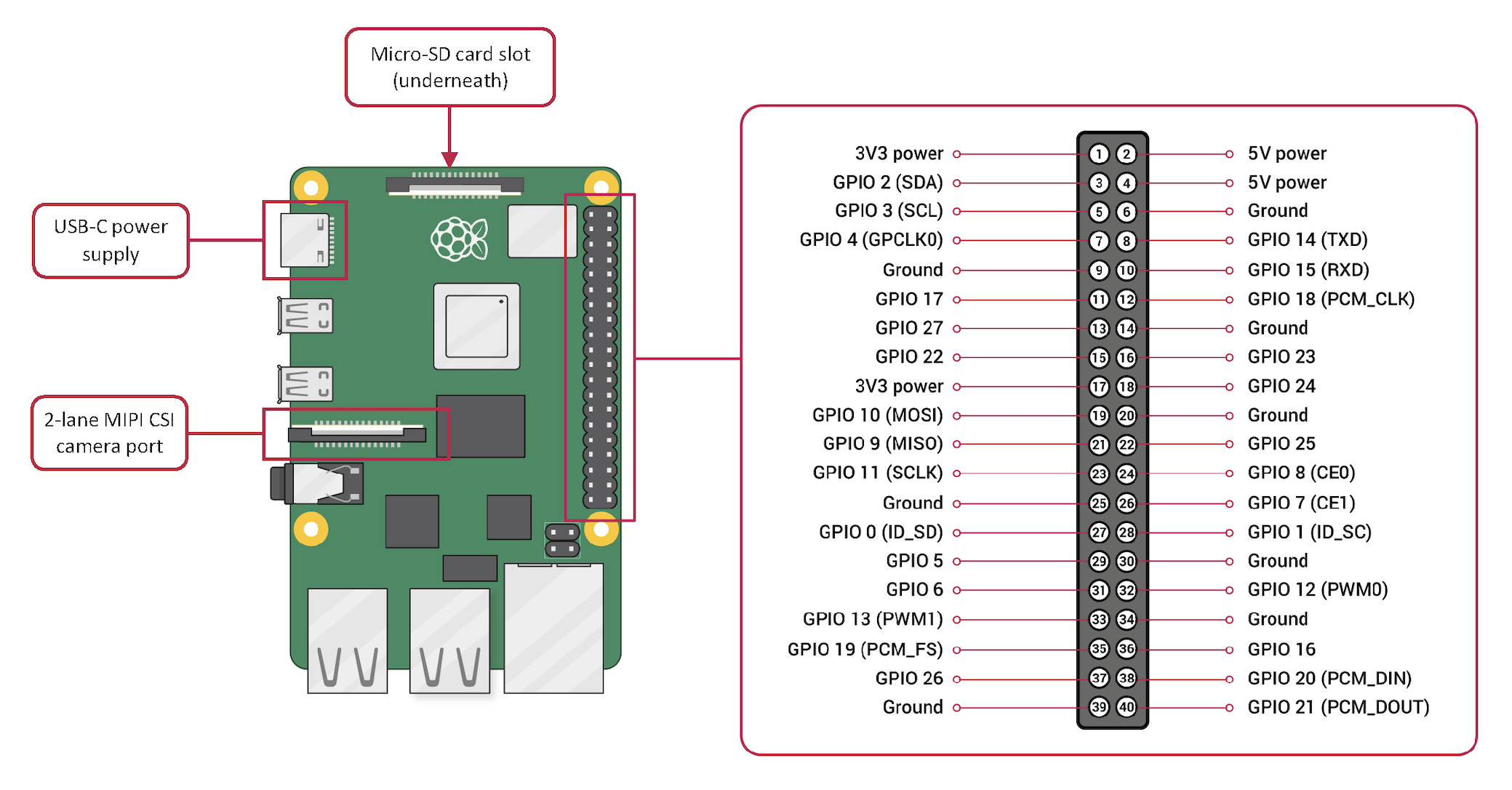

Supplement: Extended Data Figure 2-1 — Diagram of the Raspberry Pi 4B motherboard. Image source: https://github.com/raspberrypi/documentation/blob/develop/documentation/asciidoc/computers/os/using-gpio.adoc. Download Figure 2-1, TIF file. [file enu-eN-MNT-0224-22-s08.tif]

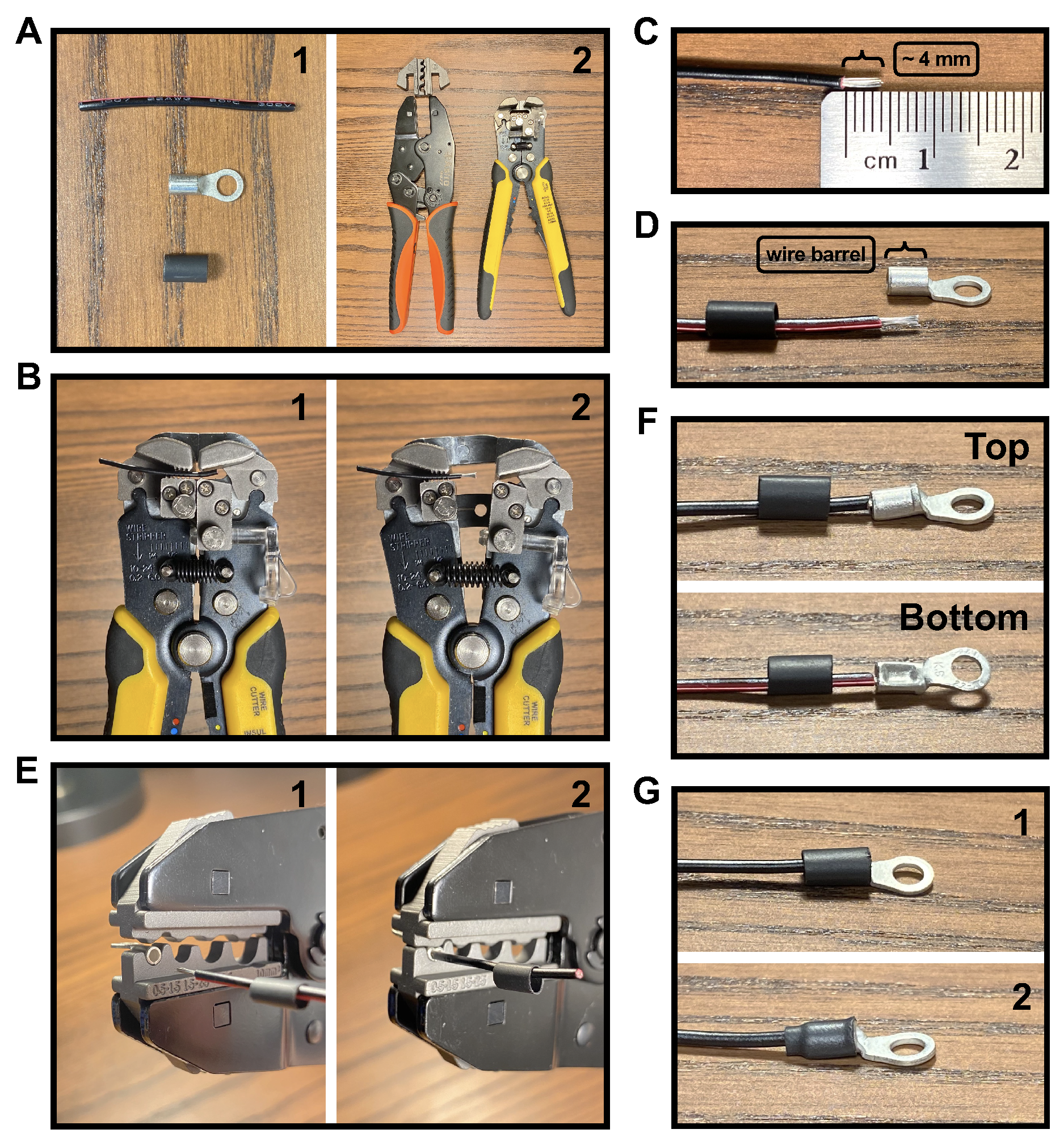

Supplement: Extended Data Figure 4-1 — Step-by-step instructions for crimping ring terminal connectors. A, Gather all essential parts: (1) electrical wire AWG22, noninsulated ring terminal connector AWG22-16 #4 stud size, heat shrink tubing 1/8”, (2) Astro crimping tool with B-Jaw for noninsulated terminals, and wire stripper. B–D, Strip the wire to expose ∼4 mm of conducting wire, comparable to the length of the wire barrel of the connector. D, Cut one short segment of 1/8” heat shrinking tubing that is long enough to cover the entire wire barrel (≥6 mm) and put it on the wire before crimping. E, Crimp the connector to the bare wire using the first die (DIN 0.5–1.5 mm2) on the B-Jaw of the Astro crimping tool. F, Make sure to visually inspect and perform a gentle pull test to confirm the crimp is successful. G, Fully cover the entire wire barrel with heat shrink tubing and use a heat gun to shrink the tubing. Download Figure 4-1, TIF file. [file enu-eN-MNT-0224-22-s09.tif]

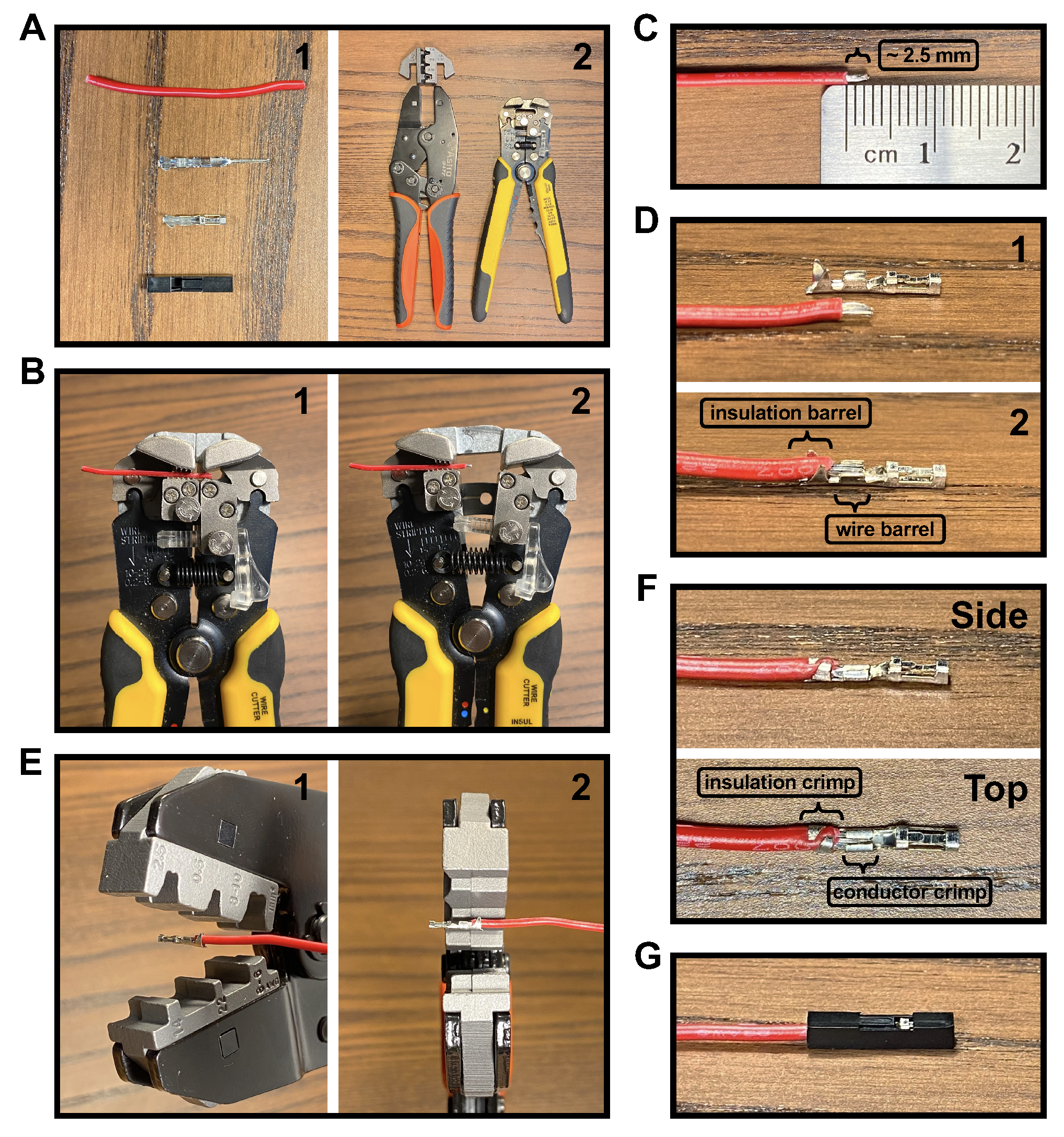

Supplement: Extended Data Figure 4-2 — Step-by-step instructions for crimp pin terminal connectors. A, Gather all essential parts: (1) electrical wire AWG22, male or female pin terminal connector, plastic housing, (2) Astro crimping tool with H-Jaw for open barrel terminals, and wire stripper. B, C, Strip the wire to expose ∼2.5 mm of conducting wire. D, Insert the stripped wire into the pin terminal connector while making sure that the bare wire falls within the wire barrel of the connector and the wire insulation is inside the insulation barrel. Gently bend the insulation barrel around the wire insulation to prevent connector or wire from moving out of place during crimping. E, Crimp the connector to the wire using the middle die (DIN 0.5 mm2) on the H-Jaw of the Astro crimping tool. Make sure that the open barrel is facing towards the “nest” of the crimping die and both wire barrel and insulation barrel are positioned in the appropriate spots of the crimping die. F, Visually inspect and perform a gentle pull test to confirm the crimp is successful. G, Insert the crimped connector into a plastic housing. Visually inspection and gentle pull test should be performed to make sure that the connector is securely housed in order to avoid issues with mating pins. Download Figure 4-2, TIF file. [file enu-eN-MNT-0224-22-s10.tif]

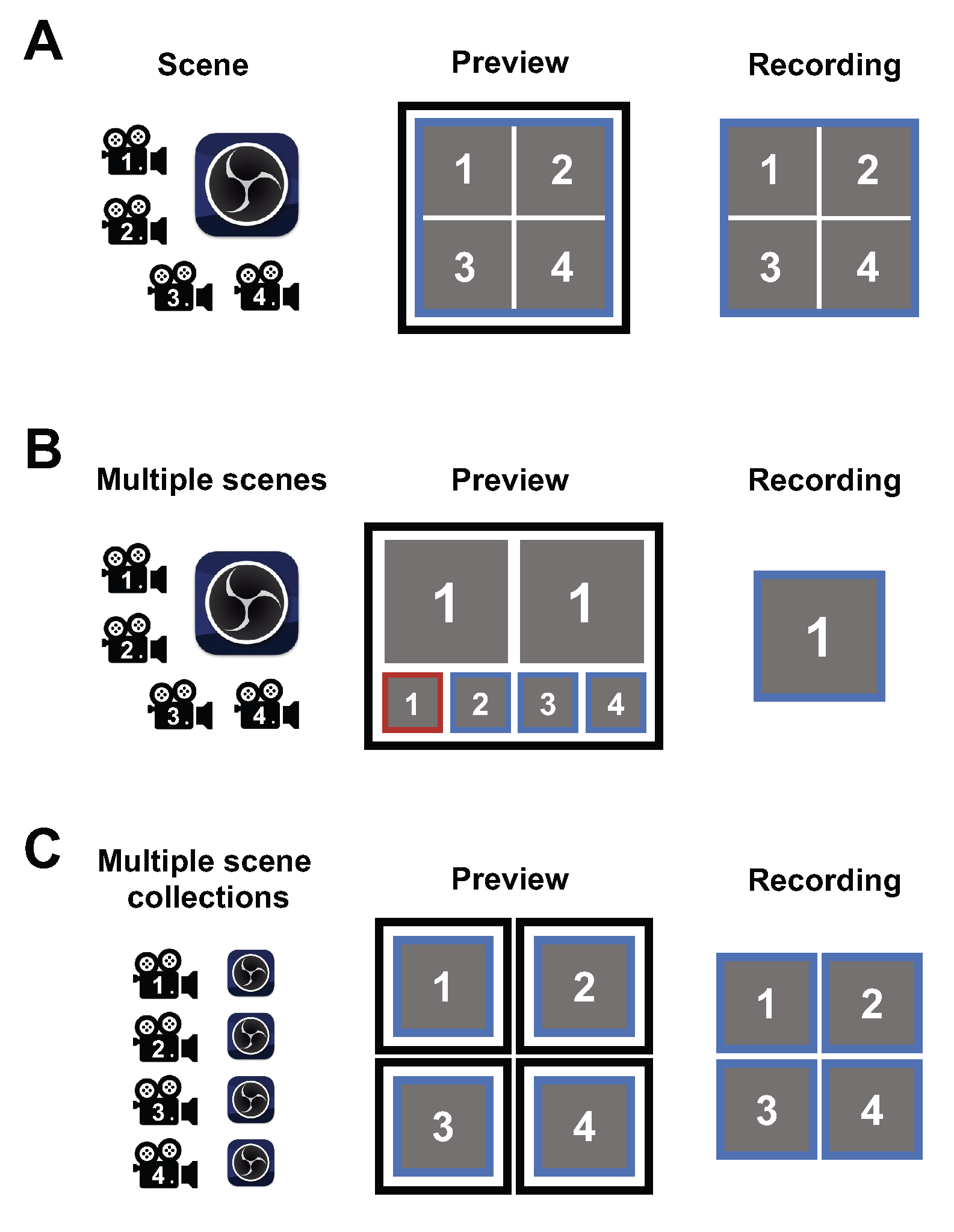

Supplement: Extended Data Figure 6-1 — Schematics showing several options to preview and/or record from multiple video sources. A, One scene with multiple video sources operating within one instance of OBS Studio recorded into a single video file. B, Multiple scenes each with their own video source operating within one instance of OBS Studio. Recording produces video from a single scene/video source. C, Multiple scene collections each with their own video source running in independent instances of OBS Studio produce independent video files. Image source: https://github.com/obsproject/obs-studio/blob/master/UI/forms/images/obs_256x256.png. Download Figure 6-1, TIF file. [file enu-eN-MNT-0224-22-s11.tif]

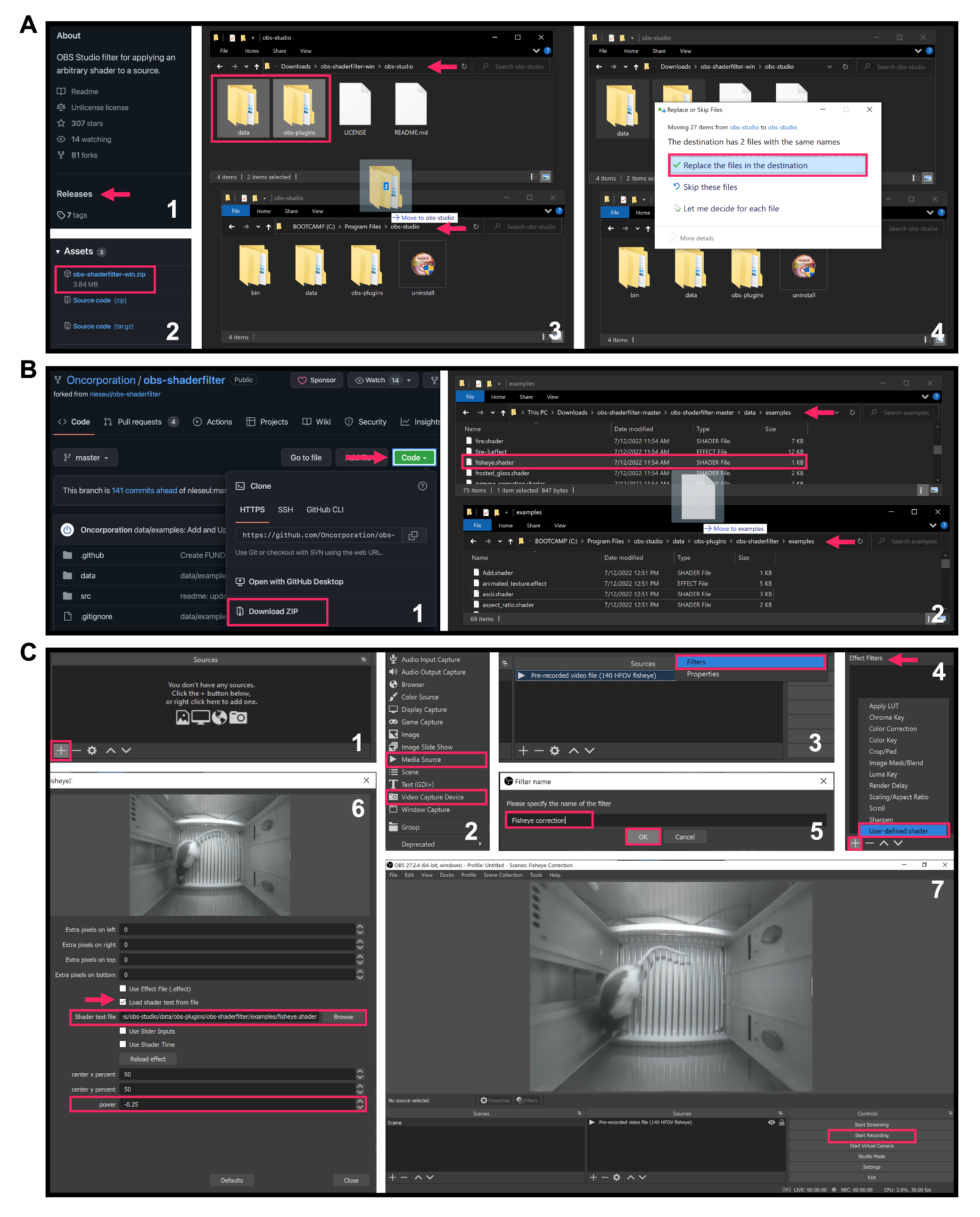

Supplement: Extended Data Figure 8-1 — Digital correction of fisheye distortion. A, Download the OBS ShaderFilter plugin from its GitHub page and install on a Windows PC equipped with OBS Studio. B, Download the “fisheye.shader” text file located in its GitHub repository. In the main OBS interface, add a prerecorded video as a “Media source” for offline fisheye correction or a camera as a “Video Capture Device” for real-time fisheye correction (C1, C2). Enable the ShaderFilter plugin and fisheye correction for each video source (C3–C7). Download Figure 8-1, TIF file. [file enu-eN-MNT-0224-22-s12.tif]

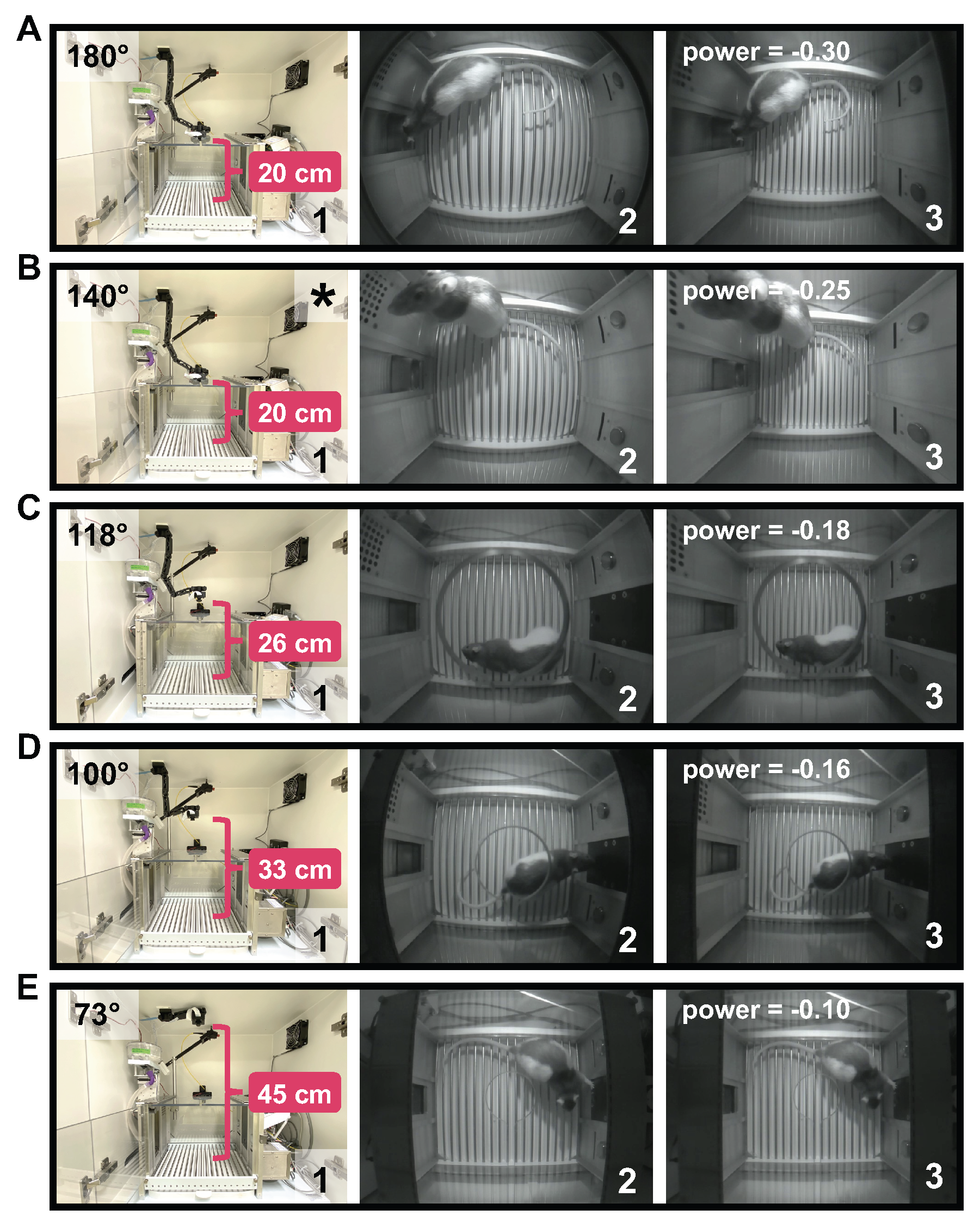

Supplement: Extended Data Figure 8-2 — Comparison of Pi USB Cam-compatible fisheye lenses in terms of object distance and fisheye correction. Pictures showing the object distance of a Pi USB Cam (1) equipped with various M12 fisheye lenses including 180° (A), 140° (B), 118° (C), 100° (D), and 73° (E) for overhead viewing in a standard operant box. Snapshots of raw videos acquired from a centered position at the corresponding object distances showing comparable captured fields (2), and the same videos after the fisheye image distortion was digitally corrected using the OBS ShaderFilter plugin (3). Power settings used are indicated on each image. *Indicates the default lens that comes with the Arducam day-night vision camera. Download Figure 8-2, TIF file. [file enu-eN-MNT-0224-22-s13.tif]

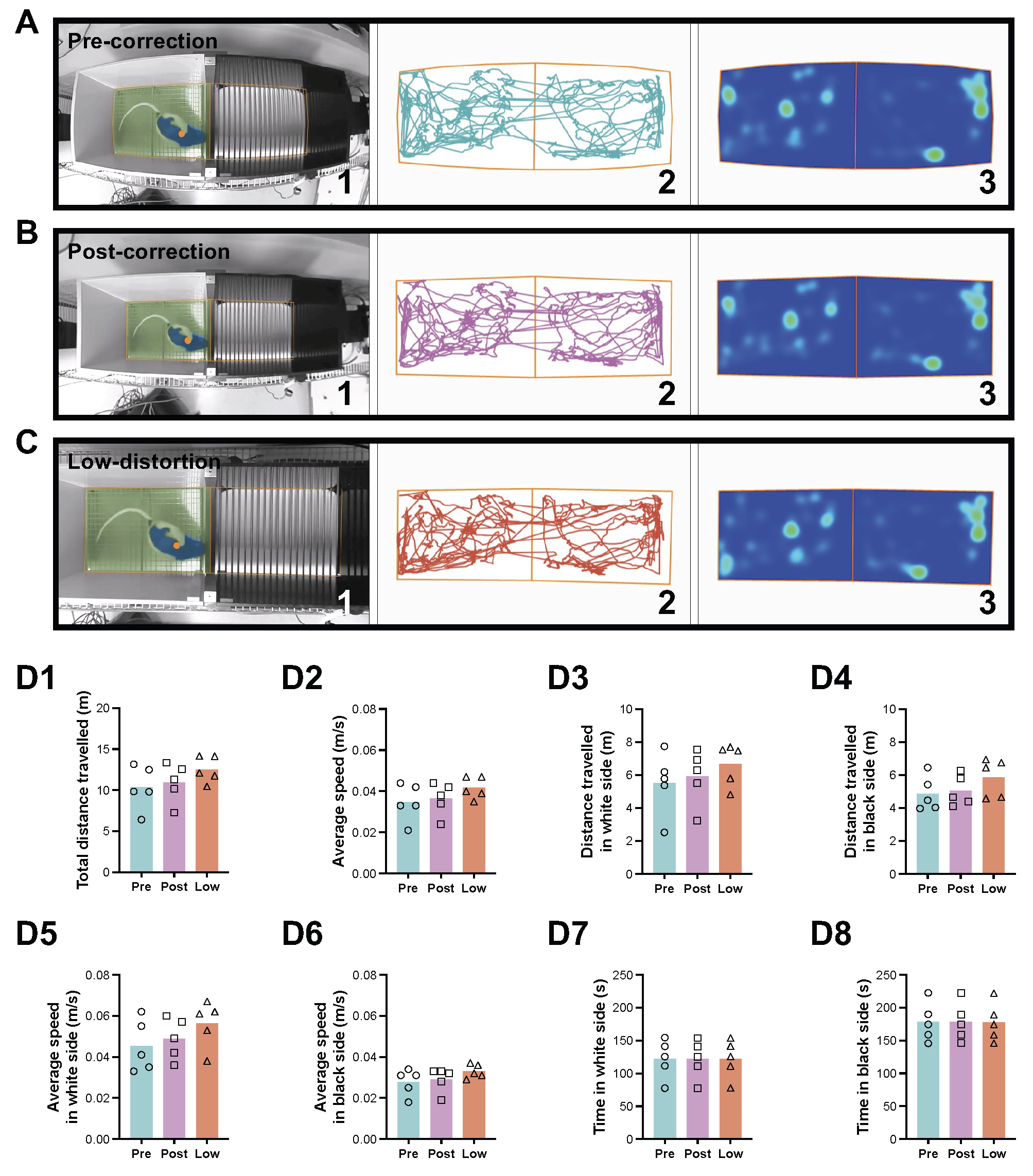

Supplement: Extended Data Figure 8-3 — Effects of fisheye distortion on position tracking and locomotor measures. Snapshots of ANY-maze position tracking (1) performed on a video acquired with a 100° HFOV fisheye lens without distortion correction (A), after digital distortion correction (B), and from a video acquired in tandem using a 70° HFOV low-distortion lens (C). Representative center-point tracking plots (2) and heatmaps (3) from the same video show similar results. D, Effects of fisheye distortion and correction on position tracking accuracy were demonstrated by comparing various locomotor activity measures, including the total distance travelled (1) and average speed (2) during the entire test duration, as well as the total distance travelled (3, 4), average speed (5, 6), and time spent (7, 8) in either side of the testing apparatus. Download Figure 8-3, TIF file. [file enu-eN-MNT-0224-22-s14.tif]
